# *RBFOX1* Dysfunction Unlocks *APOE4*‐Associated Microglial Genesis and Exacerbates Alzheimer's Pathology in Human Cerebral Organoids

**DOI:** 10.1002/exp2.70160

**Published:** 2026-04-02

**Authors:** Bowen Zhang, Changjie Shi, Jiayi Zhao, Qiuhong Hua, Houchun Zhang, Hailin Gao, Yuanyuan Qian, Jiaxue Cha, Jing Li, Jiayao Chen, Tae‐Hyung Kim, Jianhuang Xue, Yujun Hou, Ru Zhang

**Affiliations:** ^1^ Institute For Regenerative Medicine, State Key Laboratory of Cardiology and Medical Innovation Center, Shanghai East Hospital, Shanghai Key Laboratory of Signaling and Disease Research, Frontier Science Center for Stem Cell Research, School of Life Sciences and Technology Tongji University Shanghai China; ^2^ Department of Biomedical Engineering Institute For Cross‐disciplinary Studies (ICS) Sungkyunkwan University (SKKU) Suwon Gyeonggi Republic of Korea; ^3^ Key Laboratory of Spine and Spinal Cord Injury Repair and Regeneration of Ministry of Education, Tongji Hospital Affiliated to Tongji University, Frontier Science Center for Stem Cell Research, School of Life Sciences and Technology Tongji University Shanghai China

**Keywords:** Alzheimer's disease, APOE4, microglia generation, organoids, RBFOX1

## Abstract

Alzheimer's disease (AD) pathogenesis is strongly influenced by APOE4, though how cooperative genetic factors modulate this relationship remains unclear. While genomic studies have tentatively linked *RBFOX1* to AD susceptibility, its functional synergy with *APOE4* has never been experimentally defined. We engineered *APOE3* or *APOE4* isogenic human cerebral organoids with CRISPR/Cas9‐mediated *RBFOX1* knockout. Remarkably, *RBFOX1* depletion selectively triggered robust microglial generation exclusively in *APOE4* organoids. Time‐course gene expression revealed that this APOE4‐specific effect correlated with prolonged mesodermal priming during early embryoid body differentiation, creating a permissive niche for microglial lineage specification. The emergent microglia exhibited pronounced neurotoxic phenotypes, including pro‐inflammatory factor secretion, synaptic architecture remodeling, and lipid droplet accumulation in organoids. These changes coincided with aggravated Tau hyperphosphorylation and electrophysiological abnormalities, collectively mirroring multifaceted AD pathology. Our findings establish RBFOX1 as a potential AD protective factor, a critical suppressor of APOE4‐glia crosstalk, and demonstrate that its loss unleashes a microglia‐mediated neurodegenerative cascade. By developing cerebral organoids with autonomous microglial networks, we present a platform capable of modeling genotype‐dependent neuron‐glia interactions in AD, opening new avenues for mechanistic and therapeutic exploration.

## Graphical abstract

Genomic studies implicate *RBFOX1* in Alzheimer's disease (AD) risk, highlighting the need to define its role and interactions with other risk factors. Here, CRISPR/Cas9‐mediated *RBFOX1* loss in *APOE4* human cerebral organoids induces autonomous microglial genesis and accelerates AD pathology, revealing a protective role of *RBFOX1* and establishing a model for genotype‐specific neuron–glia interactions.

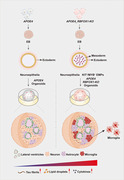

## 1 Introduction

Alzheimer's disease (AD) is a progressive neurodegenerative disorder characterized by neuronal loss, extracellular senile plaques, and intracellular neurofibrillary tangles. Familial AD (fAD), which accounts for less than 5% of all AD cases, typically presents with early‐onset symptoms between the ages of 40 and 50. Affected families have a clear family history of AD, and pathogenic gene mutations—such as those in the *APP*, *PSEN1*, or *PSEN2* genes—directly trigger the disease. In contrast, sporadic AD (sAD), representing over 95% of AD cases, predominantly affects individuals aged 65 or older [1, 2, 3, 4, 5]. A key feature is the absence of a clear familial genetic predisposition; instead, its development is associated with the combined effects of genetic and environmental risk factors. Recent genome‐wide association studies (GWAS) highlight *RBFOX1* as a potential genetic modifier of AD risk [6, 7]. For instance, *RBFOX1* single nucleotide variants (SNVs) correlate with temporal lobe volume reduction in the Alzheimer's Disease Neuroimaging Initiative (ADNI) cohort [8] and grey matter loss in mild cognitive impairment patients [9]. Despite these associations, experimental validation remains limited.

*RBFOX1* encodes an RNA splicing factor [10] critical for regulating alternative splicing and gene expression networks during brain development [11, 12]. Dysregulation of the RBFOX1 pathway has been implicated in neuropsychiatric disorders, including generalized epilepsy, intellectual disability, autism spectrum disorder, and developmental aggression‐related disorders [13, 14, 15]. Intriguingly, Raghavan et al. identified a tentative link between amyloid PET imaging signals and *RBFOX1* single nucleotide variants (SNVs) in proximity to the *APOE* locus [7]. Extensive evidence identifies *APOE4* as the strongest genetic risk factor for sAD [2, 16, 17, 18, 19, 20, 21]. However, no direct interaction between *RBFOX1* and the *APOE4* allele was established [7]. These findings underscore the need to experimentally define *RBFOX1*’s role in AD pathogenesis and its interplay with *APOE4*.

Human cerebral organoids (hCOs) derived from human embryonic stem cells (hESCs) or human induced pluripotent stem cells (hiPSCs) recapitulate key structural and functional features of the developing brain, offering powerful platforms to study neurodevelopment [22] and neurological disorders [23, 24, 25, 26, 27]. However, conventional hCOs lack endogenous microglia [28, 29]. While co‐culture strategies enable microglial integration into hCOs [30, 31, 32, 33], these chimeric models fail to replicate the autonomous self‐organization of native brain tissue, limiting their physiological relevance. Moreover, microglia play a crucial role in promoting the development of AD. Mutations and abnormal expression of risk genes in microglia, as well as their activation, can increase the likelihood of AD onset [34, 35, 36, 37]. In many AD cases, the number of microglia in the brain parenchyma is markedly increased [38]. Therefore, constructing human cerebral organoid models containing microglia is essential for studying the pathological mechanisms of AD.

In this study, we combined CRISPR/Cas9‐mediated *RBFOX1* knockout with isogenic *APOE3* or *APOE4* hCOs to investigate RBFOX1's role in AD. Strikingly, *RBFOX1* depletion induced robust microglial engraftment exclusively in *APOE4* organoids, a phenotype not observed in *APOE3* counterparts. This *APOE4*‐specific microglial expansion coincided with comprehensive AD‐like pathology, including neuroinflammation and Tau hyperphosphorylation. Importantly, our hCOs exhibited autonomous self‐organization of all cell types, including microglia, closely mimicking in vivo brain tissue architecture. This model provides a transformative tool for dissecting genotype‐specific neuron‐glia interactions in AD and accelerating therapeutic discovery.

## 2 Materials and Methods

### 2.1 Maintenance and Culture of Human Embryonic Stem Cells (H9)

H9 were cultured on plates coated with Matrigel (Corning, Cat# 354253) at 37°C and 5% CO_2_ in mTeSR1 (Stem Cell Technologies, Cat# 85850). The medium was changed daily, and the cells were split at a 1:10 ratio using 1 U mg^−1^ Dispase II (Roche, Cat# 942078001) every 5–6 days.

### 2.2 Generation of Isogenic H9 Lines

To obtain the H9 (*APOE3/3*) or H9 (*APOE4/4*) lines, we edited the H9 (*APOE3/4*) using the CRISPR/Cas9 system following the published protocol [39]. Briefly, we designed an sgRNA within 10 nucleotides from the target site corresponding to amino acid 112 of the APOE gene using the CRISPR Design tool (http://crispr.mit.edu). We also introduced single‐strand oligodeoxynucleotides (ssODN) as a repair template to convert *APOE3/4* to *APOE3/3* and *APOE4/4*. The designed sequences are in Supplementary Table S1. After co‐electroporating sgRNA plasmids and ssODN into H9 cells, the population was cultured on Matrigel‐coated dishes. After 48 h of cell culture, selection was performed using puromycin (puro) at a final concentration of 0.5 µg/mL for 48 h. The puro‐containing medium was then withdrawn, and cells were cultured for an additional 2–3 days. The cultured cells were dissociated into single cells using Accutase (500 U/mL), followed by replating of 2000 cells onto Matrigel‐coated 60 mm culture dishes to ensure sufficient intercellular distance. Cells were cultured until individual H9 clones reached sufficient size. Under stereomicroscope observation, candidate clones were identified based on: ‌(1) morphological integrity‌: exhibiting healthy growth and an undifferentiated state; ‌(2) spatial isolation‌: maintaining adequate distance from neighboring clones to ensure clonal purity. Selected clones were mechanically picked, expanded, and sequenced to confirm homozygous CRISPR/Cas9 edits. Monoclonal colonies were ultimately identified as target clones through DNA sequencing‐confirmed genotypes of either *APOE3/3* or *APOE4/4* (Supplementary Figure S1B).

### 2.3 Generation of *RBFOX1*‐knockout H9 (*APOE3* or *APOE4*) Lines

To obtain *RBFOX1*‐KO H9 (*APOE3* or *APOE4*), we designed an sgRNA using the CRISPR Design tool (http://crispr.mit.edu). The designed sequences are in Supplementary Table S1. Single‐cell clones were selected to ensure homogeneity, following the aforementioned method for obtaining homozygous *APOE3/3* and *APOE4/4* lines, and were then subsequently sequenced. Frameshift mutations were introduced successfully at the *RBFOX1* gene locus, and disruption of *RBFOX1* gene expression was confirmed by qPCR and western blot analysis (Supplementary Figures S1E–S1H).

### 2.4 Organoid Generation

**FIGURE 1:**
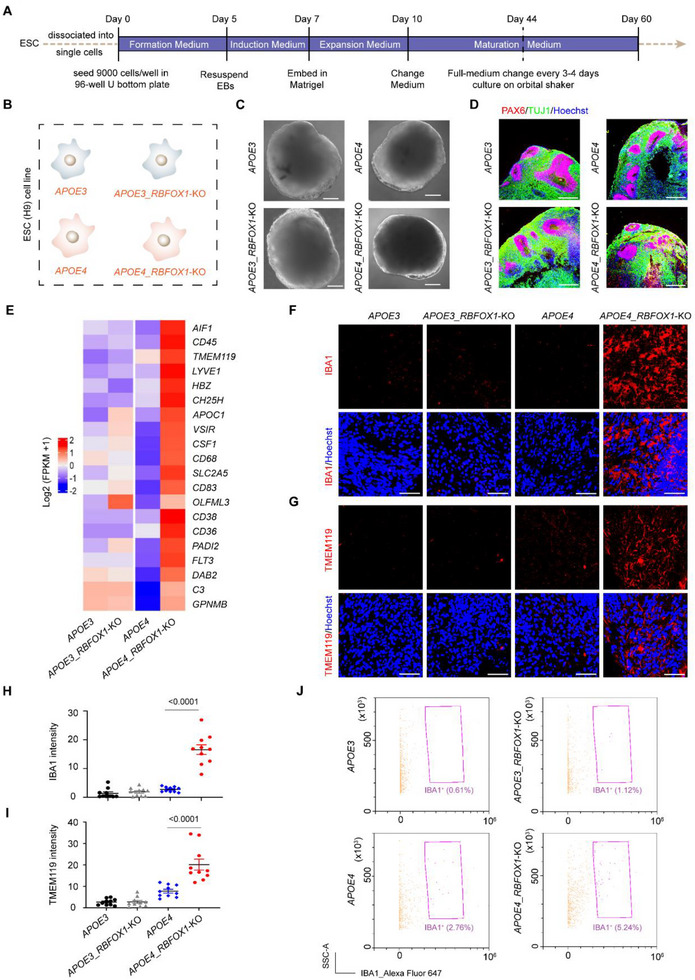
*RBFOX1* knockout triggers *APOE4*‐specific microglia generation in human cerebral organoids. (A) Schematic of generation of organoids, (B) Generation of *APOE3*, *APOE3_RBFOX1*‐KO, *APOE4*, and *APOE4_RBFOX1*‐KO H9 cell lines using CRISPR/Cas9 technology, (C) Representative bright‐field images (Day 60, Scale bars, 500 µm), (D) Immunostaining for PAX6 (red), TUJ1 (green), and DNA (Hoechst, blue) (Day 60, Scale bars, 50 µm) showed organoids well developed. (E) Gene expression heatmap of microglia signature genes in *APOE3*, *APOE3_RBFOX1*‐KO, *APOE4*, and *APOE4_RBFOX1*‐KO organoids at Day 60 from RNA‐seq data. (two biological replicates per genotype, with three organoids pooled per replicate). A pseudo‐count was used for FPKM values (FPKM + 1), log2‐transformed, and each gene was normalized in its respective row. (F, G) Representative images showing the microglia marker IBA1 and TMEM119 in organoids at Day 60, respectively. Scale bars, 50 µm, (H, I) Quantification of IBA1 and TMEM119 average fluorescence intensity shown in (F, G) respectively. Data represent the mean ± SEM and were analyzed by two‐way ANOVA. (*n* = 10; each data point represents one organoid; organoids were collected from three independent batches; image fields were randomly selected, with a minimum of eight fields analyzed per replicate), and (J) Flow cytometry analysis of IBA1 in Day 60 organoids.

Cerebral organoids were generated based on the STEMdiff Cerebral Organoid Kit (Stem Cell Technologies). A flowchart illustrating the key steps of organoid culture procedures is shown in Figure 1A. The H9 were dissociated into a single cell with 1 mL of 100 U mL^−1^ collagenase IV (Gibco, 17104‐019) at 37°C for 30 min. Then, gently aspirate the collagenase IV without disturbing the colonies and add 1 mL of 500 U mL^−1^ Accutase (Sigma‐Aldrich, A6964) for 8 min. The single cells were seeded (9000 cells/well) into 96‐well round‐bottom ultra‐low‐attachment plates (Corning, 7007) containing EB formation medium with the Rho kinase inhibitor Y‐27632 (final concentration 10 µM) (Sigma, 129830‐38‐2) to generate EBs. On day 5, EBs with diameters ranging from 400 to 600 µm, exhibiting round and smooth edges, were transferred into 24‐well ultra‐low attachment plates (Corning, 3473) in 0.5 mL induction medium, and cultured for an additional 2 days at 37°C in a CO_2_ incubator. On day 7, EBs were transferred to a pre‐cooled embedding surface, excess medium was aspirated, and 15 µL of hESC‐Qualified Matrigel (Corning, 354277) was added dropwise onto each EB. Place the plate with the embedding surface in an incubator at 37°C for 30 min to polymerize Matrigel. Then, these embedded organoids were transferred to 6‐well ultra‐low‐adherent plates (Stem Cell Technologies, 27145) with expansion medium and were cultured for 3 days. On day 10, organoids in Matrigel will develop expanded neuroepithelia, as evidenced by budding of the EB surface, and replace the medium with 3 mL/well of maturation medium, then place plates with organoids on an orbital shaker in a 37°C incubator for extended periods of culture. The medium (3 mL) was changed every 3–4 days.

### 2.5 Immunofluorescent Staining

Cells cultured on glass‐bottom well plates were fixed in 4% PFA for 15 min. Blocking and permeabilization were performed in a blocking solution consisting of 2% BSA and 0.5% Triton X‐100 for 1 h. Primary and secondary antibodies were diluted and incubated in PBST with 0.5% BSA. Cell cultures were incubated with primary antibodies at 4°C overnight, washed 3 times with PBS, and incubated with secondary antibodies for 2 h at room temperature. A defined number of microscopic fields were randomly selected to comprehensively capture fluorescence intensity across the entire human brain organoid section. Images were acquired by an Olympus IX71 inverted fluorescent microscope or an Olympus Fluoview confocal microscope, followed by calculating the average fluorescence intensity value.

Organoids were fixed in 4% PFA at 4°C overnight on a rocker, then washed 3 times for 5 min in PBS and sequentially soaked in 15% sucrose solution for 2 h, followed with 30% sucrose solution overnight at 4°C. Organoids were transferred to a capsule shell with OCT (Neg‐50; Epredia) and flash‐frozen in liquid nitrogen. The OCT samples were sectioned to 20 µm using a LEICA CM1950 cryostat. The samples were washed three times in PBST at room temperature, blocked, and permeabilized for one hour at room temperature in 2% BSA and 0.5% Triton‐X100.

Primary and secondary antibodies were diluted and incubated in the blocking solution. Primary antibodies used in this study included rabbit IBA1 (1:400, Wako, 019–19741), rabbit TMEM119 (1:400, Abcam, ab185333), rat CD68 (1:200, Invitrogen, 14‐0681‐82), mouse PSD95 (1:400, Abcam, ab2723), rabbit synapsin1 (1:400, Abcam, ab64581), mouse Tuj1 (1:400, Abcam, ab78078), mouse Pax6 (1:200, DSHB, AB_528427), mouse PHF1 (1:1000, Santa Cruz Biotechnology, SC‐81376), mouse AT8 (1:400, Pierce, mn1020), mouse 6E10 (1:400, Covence, SIG‐39300), and mouse 4G8 (1:400, Covence, SIG‐39200), LipidTOX (1:1000, Invitrogen, H34477). Cryosections were incubated with primary antibodies at 4°C overnight and washed 3 times with PBST. Secondary antibodies were species‐specific AlexaFluor‐conjugated secondary antibodies (1:500). A minimum of 8 microscopic fields were randomly selected to comprehensively capture fluorescence intensity across the entire human brain organoid section. Images were acquired by an Olympus IX71 inverted fluorescent microscope or an Olympus Fluoview confocal microscope, followed by calculating the average value.

### 2.6 Rapid Neuronal Differentiation in Essential 6 Medium (E6)

Rapid neuronal differentiation from the hESC line (H9) was performed as described previously with several modifications [40]. In brief, the human ESC line (H9) was maintained in a Matrigel‐coated 6‐well plate in mTeSR1. For neural induction, H9 colonies were dissociated into single cells and seeded in a Matrigel‐coated 6‐well plate with cell density 1 × 10^5^ ‐ 2 × 10^5^ /cm^2^. The next day, cells were confluent and were treated with LDN‐193189∙2HCl (100 nM) (Selleck, S7507) + SB431542 (10 µM) (Selleck, S1067) + XAV‐939 (2 µM) (Selleck, S1180) (LSB + X) in E6 (Gibco, A1516401) from Day 0 for 3 days. Then starting from Day 3 to Day 5, E6 with LDN193189∙2HCl (50 nM), SB431542 (5 µM), XAV‐939 (1 µM), PD0325901 (0.4 µM) (Selleck, S1036), SU5402 (2 µM) (Selleck, S7667), and DAPT (5 µM) (Selleck, S2215) (LSB + X + P/S/D) were added into the 6‐well plate. Day 5‐Day 7: N2/B27 medium was added to E6 at 1/3 (v/v); inhibitors in N2/B27 include L (250 nM) + SB (10 µM) + X (5 µM) + P (1 µM)/S (5 µM)/D (10 µM). Day 7‐Day 9: N2/B27 medium was added to E6 at 2/3 (v/v); inhibitors in N2/B27 include P (1 µM)/S (5 µM)/D (10 µM). Day 9‐Day 13: 100% NB/B27 + BDNF (20 ng mL^−1^) (PEPROTECH, 450‐02‐10UG), Dibutyryl‐cAMP (0.5 mM) (Selleck, S7858), Ascorbic acid (0.2 mM) (Sigma‐Aldrich, A4403) was used. Inhibitors used in NB/B27 include P (1 µM)/S (5 µM)/D (10 µM). All inhibitors are from Selleck unless noted.

### 2.7 MEA

MEA electrophysiological recordings were performed as described elsewhere [31, 41]. In brief, cortical organoids were plated on 6‐well MEA plates (Axion Biosystems, Atlanta, GA, USA). Recordings were performed using the Maestro MEA system and AxIS Software Spontaneous Neural Configuration (Axion Biosystems). Spikes were detected with AxIS software using an adaptive threshold crossing set to 5.5 times the standard deviation of the estimated noise for each electrode. Brightfield images were captured from each well to assess the neural density and electrode coverage over time. After recording, organoids were detached from the MEA plate and kept on the shaker in the cell culture incubator.

### 2.8 Differentiation of ESCs to Hematopoietic Progenitor Cells (HPCs)

Human ESC‐derived hematopoietic progenitors were generated using STEMdiff Hematopoietic Kit (Stem Cell Technologies). In brief, Day 1: harvest and seed human ESCs (H9) cell colonies as small aggregates in mTeSR1. Day 0–3: Change mTeSR1 to medium A and culture in an incubator. Day 3–10: change medium A to medium B on Day 3, Day 5, Day 7, and Day 10, and culture in the incubator. Day 12, harvest suspension cells. Using a 1 mL pipette tip, vigorously pipette the cells up and down and transfer the cell suspension to a collection tube. Then, add 1 mL of DMEM/F‐12 to the well and do it two times. Centrifuge the collection tube at 300 x g for 5 min at room temperature, remove and discard the supernatant, and resuspend the cell pellet in the desired medium for subsequent analyses.

### 2.9 Differentiation of HPCs to Microglia

Human HPC‐derived microglia were generated using the STEMdiff Microglia Differentiation Kit and STEMdiff Microglia Maturation Kit (Stem Cell Technologies). In brief, Day 0: Harvest HPCs. Add 1 × 10^5^ ‐ 2 × 10^5^ cells to a Matrigel‐coated 6‐well plate containing 2 mL STEMdiff Microglia Differentiation Medium. Incubate at 37°C and 5% CO_2_. Culture the cells every other day with half of the start volume of STEMdiff Microglia Differentiation Medium. Day 12: Transfer the cell suspension to a 15 mL conical tube. Centrifuge at 300 × g for 5 min. Remove supernatant until there is ∼1 mL remaining on top of the cell pellet. Add the cell suspension to a new Matrigel‐coated 6‐well plate containing 1 mL STEMdiff Microglia Differentiation Medium. Incubate at 37°C and 5% CO_2_. Culture the cells every other day for 12 days with half of the start volume of STEMdiff Microglia Differentiation Medium. Day 24: Transfer the cell suspension to a 15 mL conical tube. Centrifuge at 300 × g for 5 min. Remove supernatant until there is ∼1 mL remaining on top of the cell pellet. Add 1 × 10^6^ cells to a new coated 6‐well plate containing 1 mL STEMdiff Microglia Maturation Medium. Incubate at 37°C and 5% CO_2_. Culture the cells every other day by topping up the well with half of the start volume of STEMdiff Microglia Maturation Medium. Day 34: Microglia are collected for analyses or downstream assays.

### 2.10 Transcriptome Sequencing

In total, four different genotypes of organoids (*APOE3*, *APOE3*_*RBFOX1*‐KO, *APOE4*, and *APOE4*_*RBFOX1*‐KO) were collected on Day 60 for RNA sequencing. Each genotype was analyzed with two biological replicates, with each replicate comprising 3–4 human brain organoids. The organoid samples were sent to Berry Genomic Company (Beijing, China) for transcriptome sequencing. RNA library construction was then performed using the TruSeq RNA v2 kit, following the manufacturer's instructions. The libraries were assessed using the Agilent 4200 Bioanalyzer and sequenced on the Illumina platform using 150 bp paired‐end sequencing and then sequenced on an Illumina NovaSeq 6000 PE150 mode (Illumina, USA), producing raw reads. Post two‐stage quality filtering with BWA (v0.7.15‐r1140) and Bowtie 2 (v2.3.2), clean reads mapped to the Silva database to remove rRNA. The valid clean reads mapped to the reference genome (GRCh37.p13) (ftp://ftp.ncbi.nlm.nih.gov/genomes/all/GCF/001/433/935/GCF_001433935.1_IRGSP‐1.0/GCF_001433935.1_IRGSP‐1.0_genomic.fna.gz) using Hisat2. DEGs between groups were identified using edgeR (FDR < 0.05, |log2FC| > 1), and significantly enriched GO terms (https://www.geneontology.org/) were determined via topGO.

### 2.11 Flow Cytometry Analysis

Cells (HPC and Microglia) were collected using cold (4°C) sterile PBS. Cells were then filtered through 70 mm mesh to remove large clumps, washed with PBS 3 times: 300 × g for 5 min at room temperature, then stained (5 µL antibody/10^6^ cells) in PBS on ice for 20 min in the dark using the following antibodies: APC anti‐human CD45 Antibody clone HI30 (BioLegend, Cat# 304037), Brilliant Violet 785 anti‐human CD68 Antibody (BioLegend, Cat# 333826). After staining, microglia were washed 3 times with PBS and suspended using 300 µL PBS. Data were collected by the BD FACS Verse system and then analyzed with FlowJo V10 software.

### 2.12 Dissolve Organoids Into Single Cells for Flow Cytometry Analysis

Whole organoids were dissociated to generate single cells. Briefly, organoids were transferred to HBSS (without Ca^2+^ and Mg^2+^, −/−). And using a new, sterile razor blade, organoids were minced into small pieces (< 1 mm). Organoid pieces were then dissociated using 2.5 mL of papain (Worthington, LK003176) + DNase (Worthington, LK003170) solution. The organoid pieces were transferred into a 6‐well plate containing papain solution, and then the plate was placed on an orbital shaker at 85 rpm inside a humidified tissue culture incubator at 37°C and 5% CO_2_ for 30 mins. Use a 1 mL pipette to gently dissociate and break up minced pieces. Return the plate to the orbital shaker using the same conditions for 10 more minutes. The digestive solution was transferred into a 15 mL tube containing 5 mL HBSS^−/−^ (2–3 organoids per tube). Using a 10 mL pipette, gently pipette the minced pieces up and down 10 times. Transfer the entire volume to an empty 15 mL conical tube and wait for the debris to settle (1–3 min). Transfer the cell suspension (avoiding debris) to a new tube. Cells were filtered through a 30‐µm strainer, washed, centrifuged for 5 min at 300 g, and washed 3 times with HBSS^−/−^. Cells were then analyzed using the Trypan Blue assay and counted using the automated cell counter Countess.

### 2.13 Flow Cytometry Analysis of Microglia in Human Brain Organoids

Single cells obtained from human brain organoids were fixed with 4% formaldehyde at a ratio of 10^6^ cells per 100 µL and incubated at room temperature for 15 min, followed by three washes with PBS. Pre‐chilled cell suspensions were slowly supplemented with ice‐cold ‌methanol‌ to a final concentration of 90%, followed by permeabilization on ice for 10 min. After removing residual methanol via PBS washing, cells were resuspended in 100 µL of IBA1 antibody dilution buffer and incubated at room temperature for 1 h. Following PBS washing, cells were resuspended in 100 µL of fluorophore‐conjugated secondary antibody dilution buffer and incubated at room temperature in the dark for 30 min. After three PBS washes, cells were resuspended in 300 µL PBS and analyzed using a flow cytometer. Microglial identification was performed through two‐dimensional fluorescence analysis using the following gating strategy: (1) initial selection based on forward scatter (FSC) and side scatter (SSC) parameters to exclude debris and cell aggregates; (2) subsequent gating for IBA1^+^ populations using fluorescence thresholds established with unstained negative controls. Data acquisition and analysis were performed using CytExpert software.

### 2.14 RNA Isolation and qPCR Analysis

Total RNA was extracted from cells using TriPure Isolation Reagent (Roche, 11667165001), and the concentration of each RNA was determined using a Nanodrop 2000 spectrophotometer. cDNA was then synthesized using approximately 1 µg of RNA using M‐MLV Reverse Transcriptase (Promega, M170B) according to the manufacturer's instructions. Real‐time quantitative PCR was performed using 2x SYBR Green qPCR Master Mix low ROX (Selleck) on Stratagene Mx3000P (Agilent Technologies). The qPCR primers were purchased by Sangon Biotech, and the sequences of the primers we used were in Supplementary Table S2.

### 2.15 Phagocytosis Assays

pHrodo Red Zymosan A Bioparticles Conjugate (Thermo Fisher Scientific, cat. P35364) was rinsed and reconstituted in the Live Cell Imaging Solution (LCIS). The pHrodo Red Zymosan A Bioparticles Conjugate (100 µg mL^−1^) was added to microglia in a glass‐bottom 35 mm dish. After 2 h incubation in CO_2_ incubator, remove the pHrodo Red Zymosan A Bioparticles Conjugate from the dish and incubate microglia with Hoechst 33342 for 10 min. Aspirated Hoechst 33342 solution and washed the microglia with LCIS. Images were captured using an Olympus FluoView confocal microscope.

### 2.16 Statistical Analysis

Statistical analysis was performed using GraphPad Prism 9. For comparisons involving more than two groups two‐way ANOVA was employed as specified. Comparisons of two groups, were performed using a two‐tailed unpaired Student's *t*‐test. All differences were considered statistically significant when *p* < 0.05.

## 3 Results

### 3.1 *RBFOX1* Knockout Triggers *APOE4*‐specific Microglia Generation in Human Cerebral Organoids

Recent GWAS suggest a potential interaction between *RBFOX1* variants and *APOE4* in AD pathogenesis [7]. Thus, in this study, we aimed to assess the impact of RBFOX1 on AD pathology and to explore the relationship between *RBFOX1* and *APOE4* using the human cerebral organoid model (Figure 1A). CRISPR/Cas9 technology was employed to generate H9‐hESCs carrying the *APOE3* or *APOE4* genotype with *RBFOX1* knockout in parallel (Figure 1B, Figures S1A–S1B, and S1D‐S1H). The pluripotency and self‐renew properties of all subsequent H9 lines were not altered (Supplementary Figures S1C and S1I). The hCOs were then generated following the protocol from the Lancaster group [22] using the STEMdiff Cerebral Organoid Kit (Figure 1A). All H9 hESCs had the capacity to form 3D neural tissue, and all organoids of Day 60 exhibited a ventricular zone‐like structure surrounded by a thick layer of PAX6‐positive neural progenitor cells (Figures 1C–D) and TUJ1‐positive neuroblasts in the outer layer. A similar structure was observed in Day 44 organoids. These results indicate the successful development of hESCs to cerebral organoids with cortical‐like organization after *RBFOX1* knockout.

Given RBFOX1's role as an RNA‐binding protein (RBP) regulating RNA metabolism, we performed RNA sequencing on *APOE3*, *APOE3*_*RBFOX1*‐KO, *APOE4*, and *APOE4*_*RBFOX1*‐KO organoids. Strikingly, compared to *APOE4* controls, *APOE4_RBFOX1*‐KO organoids showed marked upregulation of microglia‐specific or macrophage‐related genes [30, 31, 42, 43, 44, 45, 46] (*AIF1*, *CD45, TMEM119*, *CSF1*, *CD68*, *GPNMB*, *APOC1*, *OLFML3*, *SLC2A5*, *C3*, *VSIR*, and *FLT3*) (Figure 1E and Supplementary Table S3). Gene Ontology (GO) analysis of differentially expressed genes (DEGs) revealed enrichment in microglia‐associated pathways, including regulation of inflammatory response and regulation of phagocytosis, in *APOE4*_*RBFOX1*‐KO organoids at Day 60 (Supplementary Figure S2A and Supplementary Table S4). In contrast, *APOE3*_*RBFOX1*‐KO organoids exhibited minimal changes in microglial gene expression (Figure 1E). Moreover, GO analysis revealed that the aforementioned microglia‐associated signaling pathways remained inactivated in *APOE3*_*RBFOX1*‐KO organoids (Figure S2A and Supplementary Table S4). Consistently, we detected the presence and spatial distribution of microglial cells across histological sections derived from *APOE4*_*RBFOX1*‐KO organoids. Immunostaining of the microglia marker IBA1 and TMEM119 in human cerebral organoids at Day 60 confirmed the presence of microglia‐like cells in the *APOE4*_*RBFOX1*‐KO organoids (Figures 1F–I and Supplementary Figure S2B). However, these microglia markers were barely detectable in the other three types of organoids. Similar results were observed when organoids of Day 44 were tested (Supplementary Figures S3A–S3B). Flow cytometry analysis of *APOE4_RBFOX1*‐KO organoids revealed that 5.24% and 5.05% of the cells were IBA1^+^ microglia on Day 60 and Day 44, respectively (Figure 1J, Supplementary Figures S2C and S3C). These percentages are comparable to the reported proportion of microglia (5%–15%) in the human brain [47, 48, 49]. qPCR analysis of Day 44 organoids demonstrated a microglia‐enriched gene expression profile in *APOE4*_*RBFOX1*‐KO samples (Supplementary Figures S3D–S3E). Together, these results indicate that *RBFOX1* knockout induces de novo microglia generation specifically in *APOE4* cerebral organoids, highlighting a genotype‐dependent interaction between *RBFOX1* and *APOE4*.

### 3.2 *APOE4*_*RBFOX1*‐KO‐induced Microglia Contributes to Inflammatory Responses

**FIGURE 2:**
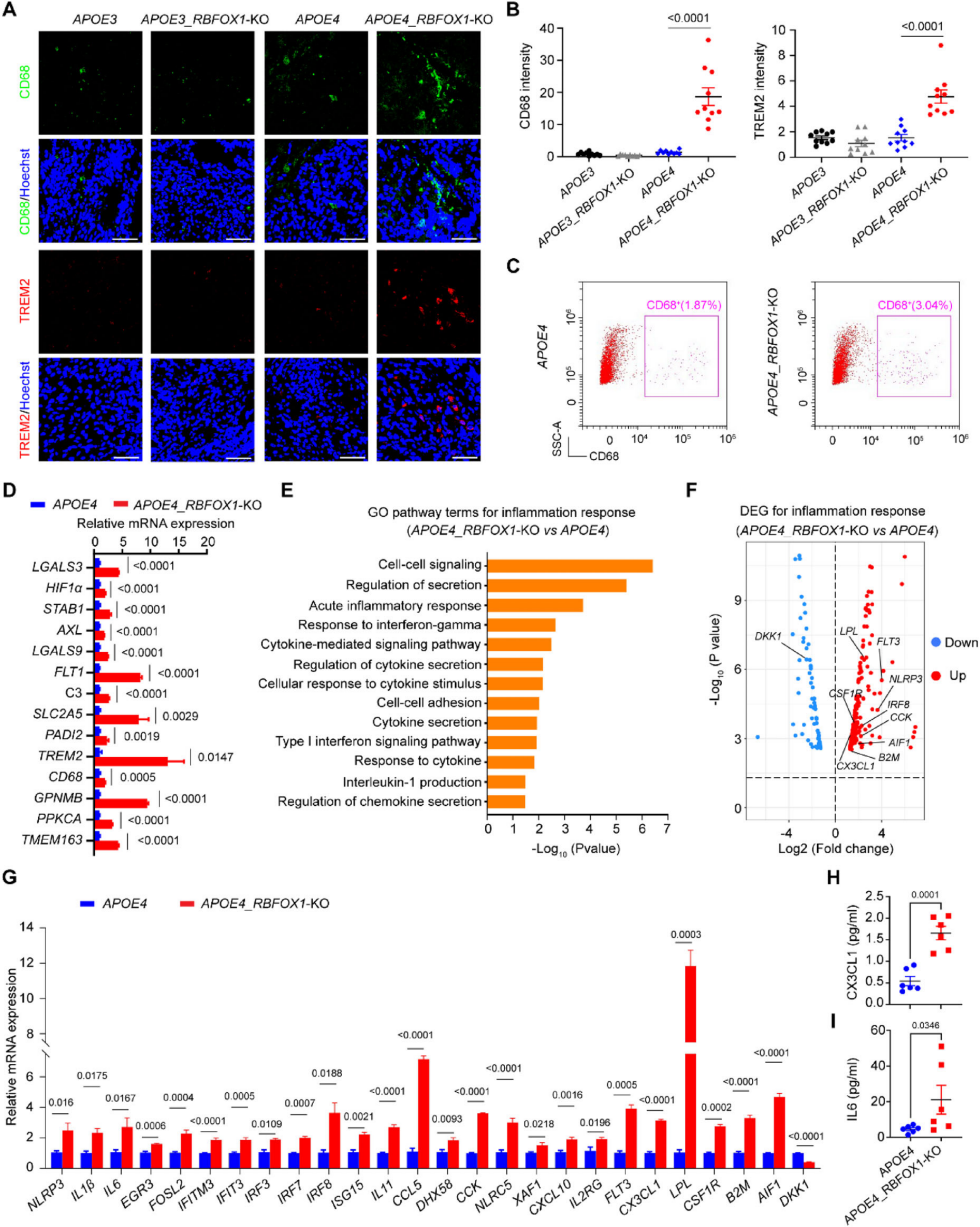
*RBFOX1*‐KO‐induced microglia contributes to inflammatory response. (A) Representative images showing the microglia marker CD68, and TREM2 in organoids at Day 60, respectively. Scale bars, 50 µm, (B) Quantification of CD68 and TREM2 average fluorescence intensity shown in (A) respectively. Data represent the mean ± SEM, and were analyzed by two‐way ANOVA. (*n* = 10; Each data point represents one organoid; organoids were collected from three independent batches; image fields were randomly selected, with a minimum of eight fields analyzed per replicate), (C) Flow cytometry analysis of CD68 in Day 60 organoids, (D) qPCR analysis for microglia‐specific DAM genes for *APOE4* and *APOE4_RBFOX1*‐KO organoids. Data represent the mean ± SEM, and were analyzed by two‐tailed unpaired Student's *t*‐test, (*n* = 3, data were collected from 3 independent experiments), (E) GO analysis for inflammation response in *APOE4_RBFOX1*‐KO organoids, (F) DEGs involved in inflammation response, (G) qPCR analysis of the fold change of inflammation factor, cytokines, and chemokines in organoids carrying the *APOE4* genotype when *RBFOX1* was knocked out. Each gene was normalized with that in *APOE4*. Data represent the mean ± SEM, and were analyzed by two‐tailed unpaired Student's *t*‐test, (*n* = 3, data were collected from 3 independent experiments), (H, I) The protein level of IL6 and CX3CL1 in organoids was tested by ELISA, and were analyzed by a two‐tailed unpaired Student's *t*‐test, (*n* = 6; data are collected from 6 independent experiments).

To elucidate the functional state of *RBFOX1*‐KO‐induced microglia in *APOE4* organoids, we analyzed markers characteristic of microglial activation. Immunofluorescence revealed robust expression of CD68 and TREM2 in *APOE4_RBFOX1*‐KO organoids, indicative of activated microglia (Figures 2A–B). Flow cytometry showed that CD68^+^ microglia accounted for 3.04% of total cells (Figure 2C), while qPCR confirmed upregulation of microglial activation‐associated genes (*LGALS3*, *HIF1α*, *STAB1*, *AXL*, *LGALS9*, *FLT1*, *C3*, *SLC2A5*, *PADI2*, *TREM2*, *CD68*, *GPNMB*, *PPKCA*, and *TMEM163*) in *APOE4* organoids with *RBFOX1* depletion (Figure 2D). Additionally, RNA sequencing data demonstrate upregulation of disease‐associated microglia (DAM) genes in *RBFOX1*‐depleted *APOE4* organoids (Supplementary Table S5), consistent with DAM phenotypes linked to neuroinflammation [50, 51].

Transcriptomic analysis of *APOE4*_*RBFOX1*‐KO organoids demonstrated significant enrichment of inflammatory pathways, including cytokine/chemokine signaling (Figure 2E and Supplementary Table S6). Key classical microglia markers (*AIF1, CSF1R, CX3CR1*, and *IRF8*) and pro‐inflammatory genes (*NLRP3, LPL*, *B2M, FLT3, and CCK*) were markedly upregulated in *APOE4*_*RBFOX1*‐KO organoids (Figure 2F and Supplementary Table S6). To dissect cell type‐specific contributions, we differentiated *APOE4_RBFOX1*‐KO hESCs into neurons, astrocytes, and microglia in 2D cultures (Supplementary Figures S4A–S4C). *RBFOX1* knockout selectively elevated inflammatory mediators (*CCL5*, *CXCL10*, *IFITM3*, *IRF3*, *NLRC5*, and *IL2RG*) in organoids and microglia but not in neurons or astrocytes (Supplementary Figures S4D–S4I), implicating that microglia may be the primary drivers of the inflammation response in *APOE4*_*RBFOX1*‐KO organoids. Further qPCR profiling revealed broad upregulation of pro‐inflammatory factors (*NLRP3, IL1β, IL6, EGR3, FOSL2, IFITM3, IFIT3, IRF3, IRF7, ISG15, IL11, CCL5, DHX58, NLRC5, XAF1, CXCL10, IL2RG*, and *CX3CL1*) in *APOE4*_*RBFOX1*‐KO organoids compared to *APOE4* organoids (Figure 2G). Additionally, ELISA results showed a trend toward an increased level of IL6 in *APOE4*_*RBFOX1*‐KO organoids (Figure 2I). CX3CL1/CX3CR1 axis activation, a key inflammatory pathway in neurodegeneration [52, 53, 54, 55, 56, 57], was confirmed by increased CX3CL1 mRNA and protein expression (Figures 2G–H). Together, these results demonstrated that *RBFOX1*‐KO‐induced DAM‐like microglia exacerbate neuroinflammation in *APOE4* organoids.

### 3.3 *RBFOX1* deletion Prolongs Mesodermal Priming to Enable Microglial Lineage Specification

Microglia in the human brain originate from erythro‐myeloid precursors in the yolk sac during embryonic development and later migrate and reside in the central nervous system [58, 59, 60]. Conventional cerebral organoids, generated via neuroectoderm‐directed differentiation, lack mesodermal lineages [28, 29]. The emergence of microglia in *RBFOX1*‐KO organoids prompted us to investigate whether RBFOX1 regulates mesoderm‐to‐microglia lineage commitment.

**FIGURE 3:**
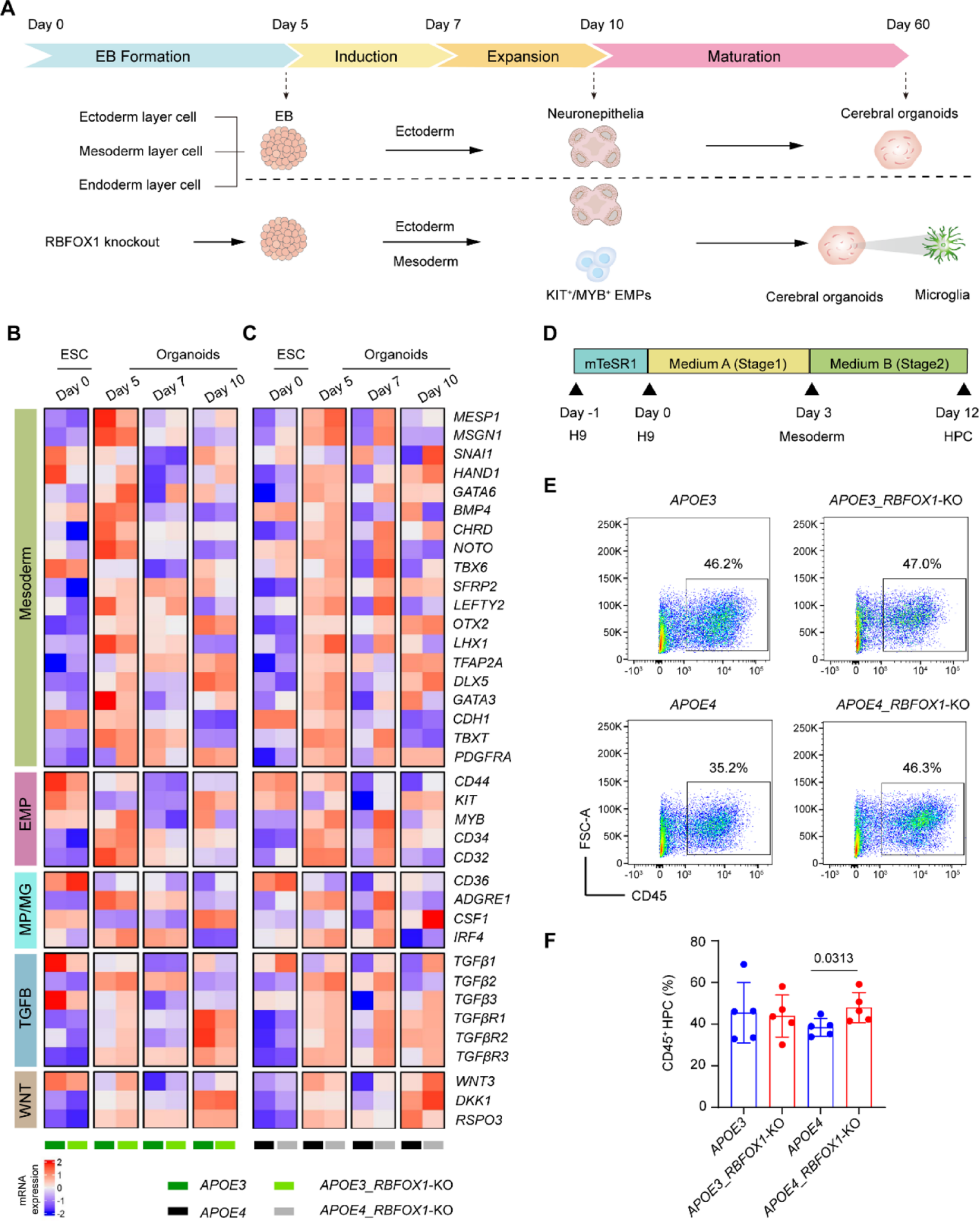
*RBFOX1* deletion prolongs mesodermal priming to enable microglial lineage specification. (A) Schematic of mesoderm development schedule in human cerebral organoids, (B, C) A heatmap of qPCR results showing marker gene expression of mesoderm, EMP, MP, microglia, TGFβ pathway and WNT pathway regulatory molecules in Day 0, Day 5, Day 7, and Day 10 *APOE3_RBFOX1*‐KO organoids and *APOE3* organoids (B), or in *APOE4_RBFOX1*‐KO organoids and *APOE4* organoids, (C) Each gene was normalized with that in *APOE3* organoids at Day 0. The significance analysis is summarized in Supplementary Table S7, (D) Schematic of generation of HPC from H9 cell lines, (E) Flow cytometry analysis demonstrated the effect of *RBFOX1*‐KO on the development of HPC (CD45^+^), and (F) Diagram graph showing the average percentage of CD45^+^ cells in FACS analysis. Data represent mean ± SEM of four different batches, and were analyzed by two‐way ANOVA. (*n* = 5; data are collected from 5 independent experiments.).

In our organoid generation protocol, the embryoid body (EB) formation, which can give rise to progenitors from the endoderm, ectoderm, and mesoderm [42, 61, 62], was initiated by Day 5. This was followed by neuroepithelia induction on Day 6 and Day 7 and continued expansion until Day 10, simulating the early stages of human brain development [63] (Figure 3A). qPCR revealed transient mesoderm‐specific gene expression peaking at Day 5 and subsequently declining from Day 7 onwards, aligning with the timeline of mesoderm formation and neuroepithelia induction observed in all hESC lines except the *APOE4*_*RBFOX1*‐KO line (Figures 3B–C and Supplementary Table S7). Strikingly, *APOE4*_*RBFOX1*‐KO organoids sustained mesoderm gene expression until Day 7, with delayed downregulation by Day 10, indicating a prolonged mesoderm induction phase that could potentially support enhanced microglia development (Figure 3C). Furthermore, genes associated with erythromyeloid progenitors (EMPs) and myeloid progenitors (MPs) exhibited prolonged induction exclusively in the *APOE4_RBFOX1*‐KO organoids. These results suggested that *RBFOX1* knockout extended mesodermal priming enables microglial specification.

The TGFβ signaling pathways play a crucial role in mesoderm development and microglia generation [64, 65, 66]. We assessed whether *RBFOX1* knockout influenced the TGFβ pathway. As shown in Figure 3C, *RBFOX1* knockout resulted in the sustained expression of *TGFβ1, TGFβ2, TGFβ3*, and their receptors, *TGFβR1, TGFβR2*, and *TGFβR3*, up to Day 7. To directly validate RBFOX1's influence on TGFβ signaling, we rescued RBFOX1 expression in *APOE4*_*RBFOX1‐*KO organoids (Supplementary Figure S5). Specifically, RBFOX1 was introduced into the *APOE4_RBFOX1‐* KO cell line, and western blot analysis confirmed successful rescue of RBFOX1 expression (Supplementary Figure S5C). These cells were then further cultured to generate organoids. On Day 5 and Day 7, we observed that restoring RBFOX1 expression led to a significant reduction in the expression of key TGFβ signaling molecules, with levels comparable to those in *APOE4* organoids (Supplementary Figures S5D–S5E). This result demonstrates that RBFOX1 regulates the TGFβ pathway, a mechanism that contributes to the regulation of microglial production. Additionally, genes involved in the WNT pathway [64, 65], including *WNT3* and *DKK1*, were significantly upregulated in *APOE4*_*RBFOX1*‐KO organoids compared with *APOE4* organoids. These findings suggest that *RBFOX1* knockout promotes mesoderm development in *APOE4* organoids through multiple signaling pathways.

To further investigate the role of RBFOX1 in the development of the progenitor cells for microglia, we differentiated H9 hESCs into hematopoietic progenitor cells (HPCs) using a 2D culture system (Figure 3D). Flow cytometry analysis for CD45 showed that when *RBFOX1* was knocked out in H9 with the *APOE4* genotype, the percentage of CD45^+^ HPCs increased (Figures 3E–F). However, *RBFOX1* knockout in H9 with *APOE3* genotype did not affect the production rate of HPCs. Therefore, we speculated that *RBFOX1* deletion prolongs mesodermal priming via TGFβ/WNT signaling, promoting EMP/MP differentiation into microglia specifically in *APOE4* organoids.

### 3.4 *RBFOX1* Knockout Exacerbates Tau Hyperphosphorylation in *APOE4* Organoids

**FIGURE 4:**
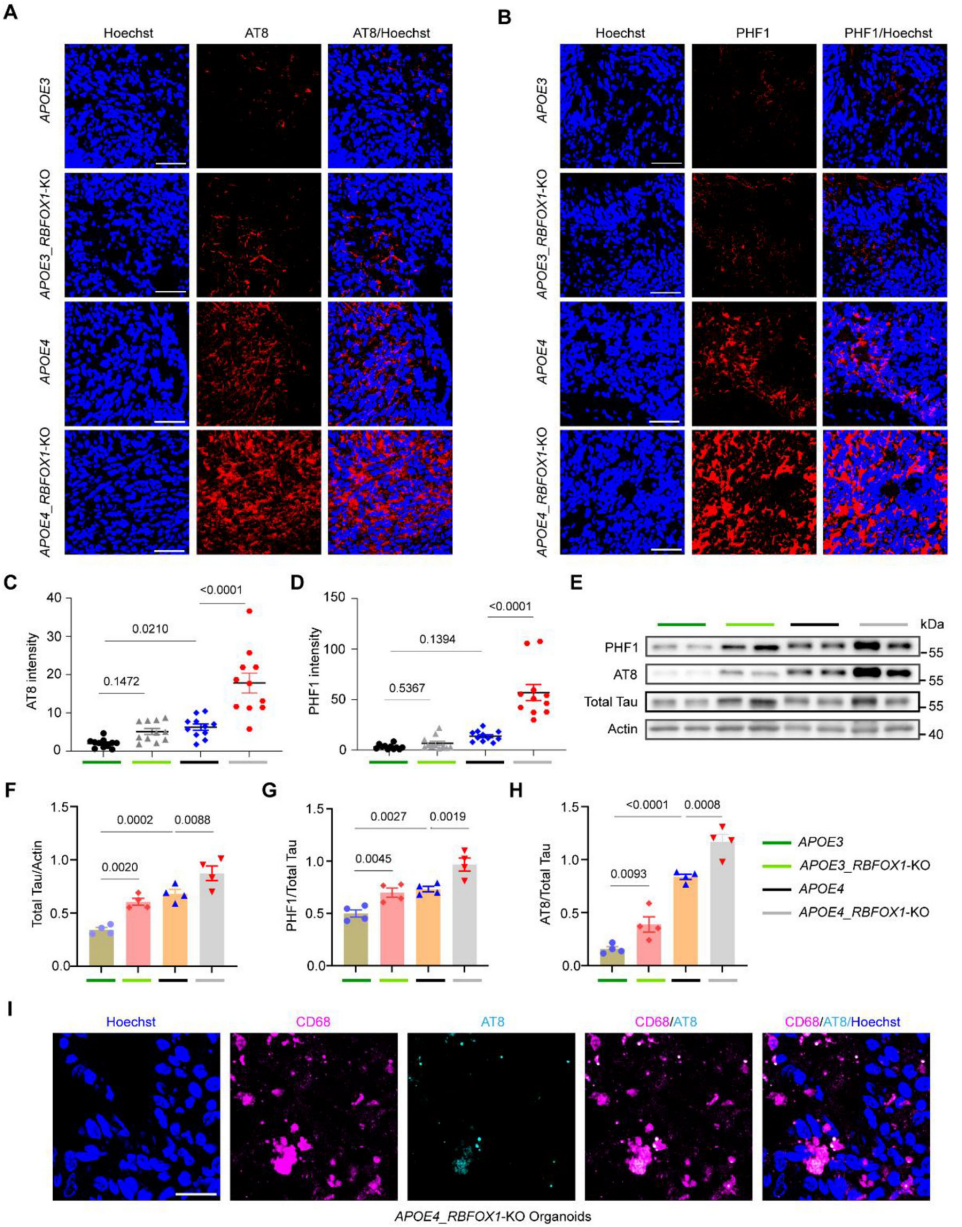
*RBFOX1* knockout exacerbates tau hyperphosphorylation in *APOE4* organoids. (A–B) Representative images showing p‐tau staining by AT8 antibody and PHF1 antibody in organoids at Day 60, respectively. Scale bars, 50 µm, (C–D) Quantification of the average fluorescence intensity in A and B. Data represent the mean ± SEM, and were analyzed by two‐way ANOVA. (*n* = 11; each data point represents one organoid; organoids were collected from three independent batches;image fields were randomly selected, with a minimum of eight fields analyzed per replicate), (E) Western Blot analysis for p‐tau, (F, G, H) Quantification of the Total tau, AT8, and PHF1 detected in (E). Data represent the mean ± SEM, and were analyzed by two‐way ANOVA (*n* = 4, data are collected from 4 independent experiments). (I) Immunofluorescent co‐staining of microglia (labeled with CD68) and p‐tau (AT8) in the organoids. Scale bars, 25 µm.

RBFOX1 is implicated in neuronal function and neurogenesis. To explore its impact on AD pathology, we assessed tau‐related pathology in hCOs with RBFOX1 depletion. Immunostaining with AT8 (targeting p‐Tau at Ser202/Thr205) and PHF1 (targeting p‐Tau at Ser396/Ser404) antibodies revealed elevated phosphorylated Tau (p‐Tau) levels in *APOE4* organoids compared to *APOE3* controls at Day 60 (Figures 4A–D), consistent with previous findings [16, 21, 67]. *RBFOX1* knockout further amplified p‐Tau levels, with a pronounced effect in *APOE4* organoids. These results were corroborated by western blot analysis using AT8 or PHF1 antibodies (Figures 4E,G, and H). Additionally, total Tau expression was higher in *APOE4* organoids and increased further upon *RBFOX1* depletion (Figures 4E–F). Collectively, *RBFOX1* knockout exacerbates the tauopathy in APOE4 organoids.

To further examine the role of RBFOX1 in Tau phosphorylation in neurons, we differentiated H9 cells into neurons using a 2D system. As detected by AT8 and PHF1 staining, *APOE4* neurons exhibited higher levels of p‐Tau than *APOE3*. In *APOE3* neurons, *RBFOX1* knockout led to increased p‐Tau levels (Figure S6A–S6D), but no significant difference was observed between the *APOE4* and *APOE4*_*RBFOX1*‐KO neurons. This suggests that interaction between neurons and other glial cell types may play a crucial role in triggering tauopathy in organoids. Consistently, a pHrodo assay on 2D‐derived microglia showed that *RBFOX1* knockout enhanced phagocytosis in the *APOE4* genetic background (Figure S6E–S6F). Notably, double immunostaining for CD68 and AT8 in *APOE4*_*RBFOX1*‐KO organoids revealed that microglia colocalized with p‐Tau, suggesting potential involvement in the phagocytosis of Tau tangles (Figure 4I). Together, these results demonstrate that *RBFOX1* deletion exacerbated p‐Tau in both neurons and cerebral organoids and, in combination with *APOE4*, produces synergistic effects on tauopathy.

We also examined an additional hallmark of AD pathology: Aβ deposition. As expected, compared to *APOE3*, an increased level of Aβ deposits was observed in H9‐derived organoids or neurons carrying the *APOE4* allele (Figures S7A–S7D). Notably, *RBFOX1* knockout did not alter Aβ deposition in organoids (Supplementary Figures S7A–S7D) or neurons (Supplementary Figures S7E–S7F) of all *APOE* genotypes, highlighting its specific role in tau pathology. These data establish RBFOX1 as a protective factor against *APOE4*‐driven tau hyperphosphorylation, operating through microglia‐dependent mechanisms.

### 3.5 The Microglia‐containing *APOE4*_*RBFOX1*‐KO Organoids Exhibit Lipid Accumulation

**FIGURE 5:**
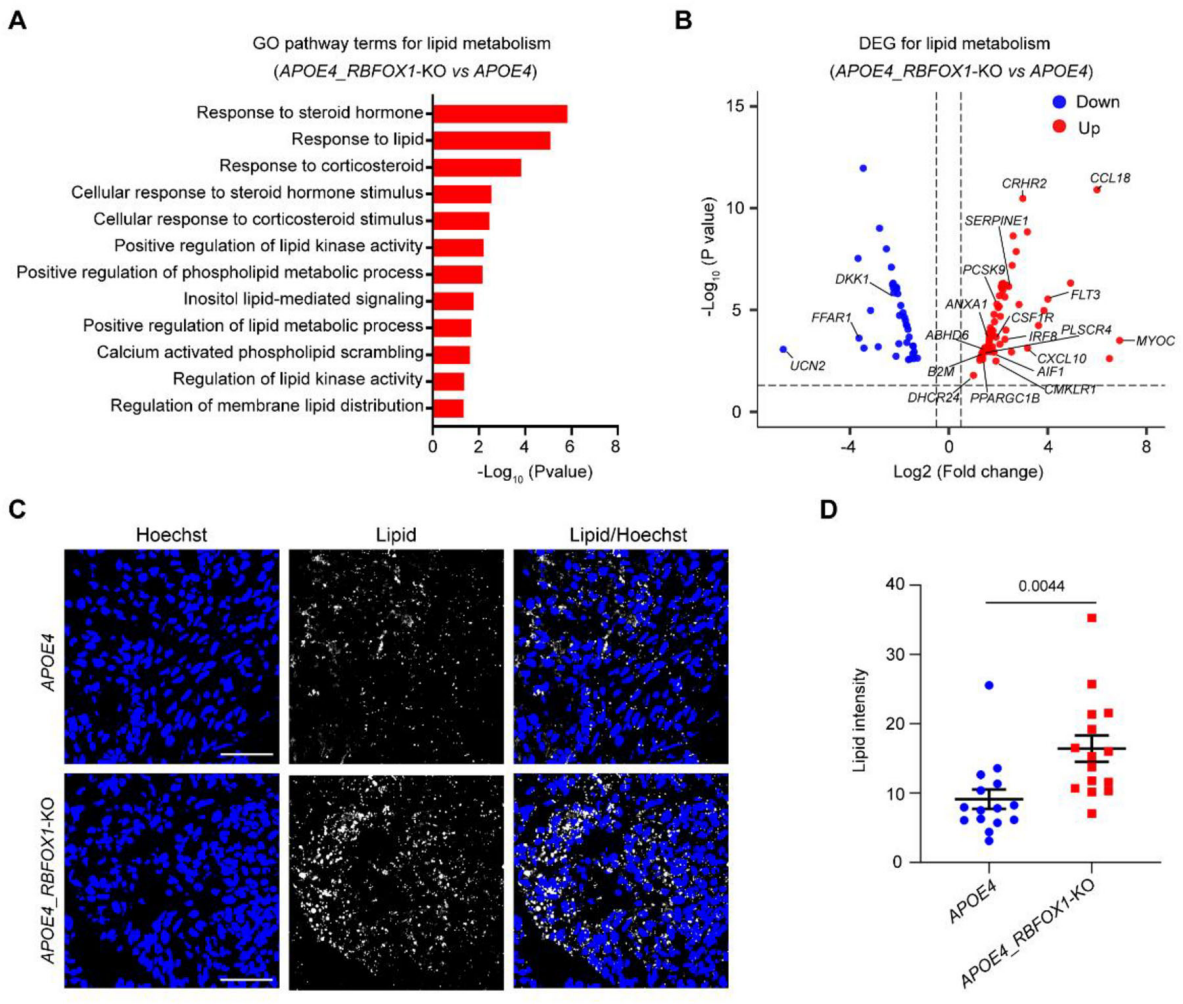
The microglia‐containing *APOE4*_*RBFOX1‐*KO organoids exhibit features of lipid accumulation. (A) GO analysis for lipid metabolism in *APOE4_RBFOX1*‐KO organoids, (B) DEGs involved in lipid metabolism, (C) Representative images showing lipid accumulation using LipidTOX Neutral Lipid Stains in organoids at Day 60. Scale bars, 50 µm, (D) Quantification of the average fluorescence intensity of the lipid stains in (C). Data represent the mean ± SEM, and were analyzed by two‐tailed unpaired Student's *t*‐test, (*n* = 15; each data point represents one organoid; organoids were collected from three independent batches;image fields were randomly selected, with a minimum of eight fields analyzed per replicate).

Lipid metabolism is closely linked to AD pathology. Disruption of lipid homeostasis in microglia has been associated with various neurological diseases [68, 69, 70, 71]. Given the strong relationship between lipid metabolism, AD, and microglia, we investigated lipid homeostasis in *APOE4_RBFOX1*‐KO organoids. RNA‐seq analysis revealed upregulation of lipid/steroid metabolism pathways in *APOE4_RBFOX1*‐KO organoids compared to *APOE4* organoids (Figure 5A and Supplementary Table S6). DEGs between the two organoid types highlighted changes in lipid metabolism‐related genes, such as *PCSK9*, *CMKLR1, DHCR24, CRHR2*, *MYOC*, *PPARGC1B*, *SERPINE1, DDK1, UCN2*, and *FFAR1* [72, 73, 74, 75, 76, 77]. Microglia‐specific genes (*AIF1*, *CSF1R*, *B2M*, *IRF8*, and *FLT3*) and inflammation‐related genes (*CCL18* and *CXCL10*) were also identified (Figure 5B and Supplementary Table S6). Subsequent lipid staining experiments showed pronounced lipid droplet accumulation in *APOE4*_*RBFOX1*‐KO organoids compared to *APOE4* controls (Figures 5C–D). These results suggest that lipid metabolism dysfunction is exacerbated in *APOE4*_*RBFOX1*‐KO organoids, and the presence of microglia, along with the activation of inflammatory responses, may further intensify lipid accumulation in this *APOE4* AD organoid model. Mechanistically, the RNA‐seq data (Figures 5A–B) showed that *RBFOX1* deficiency upregulated key microglial genes linked to lipid metabolism and inflammation: *CMKLR1*, a microglial surface receptor bound to Chemerin adipokine, modulates tau hyperphosphorylation [73] and inflammatory responses [78, 79]; *DHCR24*, a crucial enzyme regulating cholesterol biosynthesis, affects tau hyperphosphorylation through the activation of lipid rafts/caveolae‐dependent RAS/MEK/ERK signaling [74]; *PCSK9*, which encodes a major regulator of the low‐density lipoprotein receptor (LDLR), induces proinflammatory cytokines in macrophages [80] and lipid alteration [72]. These findings suggest a potential feedforward loop connecting microglial activation, lipid dysregulation, and tau pathology in *APOE4_RBFOX1*‐KO organoids. By integrating three AD hallmarks—tauopathy, neuroinflammation, and lipid accumulation—this model recapitulates multifaceted AD pathophysiology.

### 3.6 *RBFOX1*‐KO Remodels Synaptic Architecture in Microglia‐containing Organoids

**FIGURE 6:**
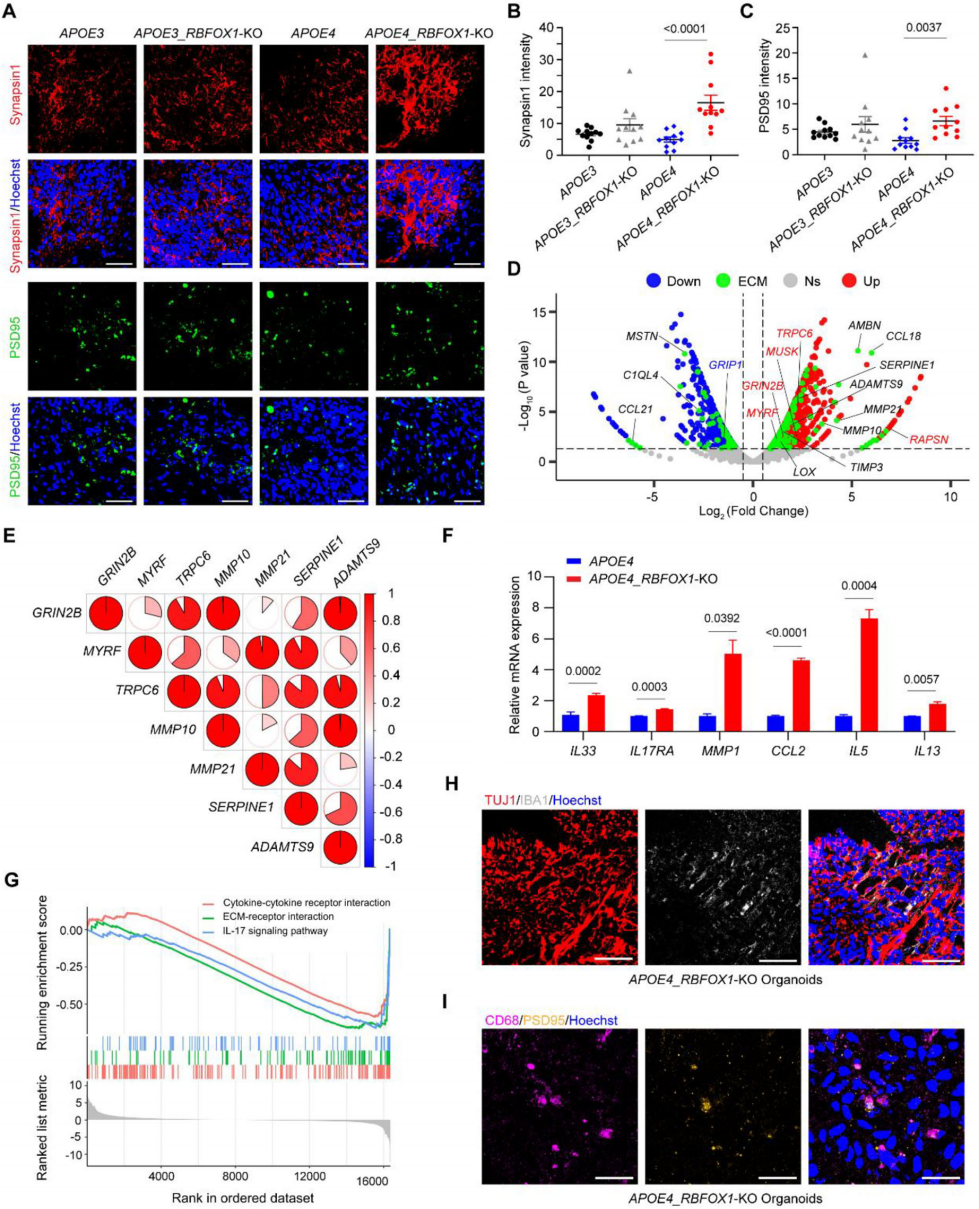
*RBFOX1‐*KO remodels synaptic architecture in microglia‐containing organoids. (A) Representative images showing synapses marker Synapsin 1 and PSD95 in organoids at Day 60, respectively. Scale bars, 50 µm, (B, C) Quantification of average fluorescence intensity in (A). Data represent the mean ± SEM, and were analyzed by two‐way ANOVA. (*n* = 11 organoids; each data point represents one organoid; organoids were collected from three independent batches; image fields were randomly selected, with a minimum of eight fields analyzed per replicate), (D) Volcano plot of differentially expressed genes after *RBFOX1* knockout in *APOE4* genetic background (*p* < 0.05) (*APOE4_RBFOX1*‐KO vs. *APOE4* organoids). Blue, down‐regulated genes; Red, up‐regulated genes; Green, ECM and ECM‐associated genes; Abbreviation: Ns, No significant, (E) Correlation analysis of synaptic remodeling genes (*GRIN2B*, *MYRF*, and *TRPC6*) and ECM‐associated genes (*MMP10*, *MMP21*, *SERPINE1*, and *ADAMTS9*), (F) The expression of *IL33* and IL17 signaling pathway genes in Day 60 organoids by qPCR analysis. Data represent the mean ± SEM, and were analyzed by two‐tailed unpaired Students *t*‐test (*n* = 3, data were collected from 3 independent experiments),(G) GSEA (KEGG) analysis of RNA‐seq data exhibiting Top3 signaling pathway in *APOE4_RBFOX1*‐KO vs *APOE4* organoids at Day 60. (H) Co‐staining of IBA1 and TUJ1 in Day 60 organoids in *APOE4_RBFOX1*‐KO organoids at Day 60. Scale bars, 50 µm, and (I) Co‐staining of CD68 and PSD95 in Day 60 organoids in *APOE4_RBFOX1*‐KO organoids at Day 60. Scale bars, 25 µm.

To evaluate synaptic alterations in *APOE4_RBFOX1*‐KO organoids, we analyzed pre‐ and post‐synaptic markers. A slight decrease in presynaptic (Synapsin1) and postsynaptic (PSD95) signals was observed in *APOE4* organoids compared to *APOE3* organoids. Strikingly, *RBFOX1* knockout significantly increased Synapsin1 and PSD95 levels in *APOE4* organoids, while no noticeable changes were observed in *APOE3* organoids (Figures 6A–C). Furthermore, RNA‐Seq data supported the role of RBFOX1 in synapse formation (*GRIN2B* [81]) and synaptic remodeling (*TRPC6* [82] and *MYRF* [83]) (Figure 6D). Additionally, the upregulation of *MUSK* and *RAPSN*, the key proteins for neuromuscular junctions [84], also indicated the effect of RBFOX1 in synapse differentiation and formation.

The neural extracellular matrix (ECM) is an essential component of synapses, providing a scaffold for synaptic structures [85, 86], supporting their stabilization and shaping, and regulating synaptic plasticity [87, 88, 89]. We, therefore, studied the expression of ECM and ECM‐associated genes in *APOE4* and *APOE4*_*RBFOX1*‐KO organoids. *RBFOX1* knockout altered 212 ECM‐associated genes (Figure 6D, green dots) in *APOE4* organoids, including marked upregulation of five ECM regulator genes (*ADAMTS9*, *MMP10*, *MMP21*, *SERPINE1*, and *LOX*). These ECM regulator genes showed strong correlates with synaptic genes (*GRIN2B*, *TRPC6*, and *MYRF*) (Figure 6E), suggesting coordinated ECM‐synapse remodeling.

The ECM and the immune system are mutually dependent [90]. In our study, we found that *CXCL10* (Figure 2H), a gene encoding a chemokine that interacts with ECM glycosaminoglycans [91], and *IL33* (Figure 6F), a gene encoding cytokine that promotes microglial‐mediated ECM engulfment and synaptic plasticity [92], were significantly upregulated in *APOE4_RBFOX1*‐KO organoids compared to *APOE4* organoids. The GSEA and KEGG pathway analyses identified three major enriched pathways: cytokine‐cytokine receptor interaction, ECM‐receptor interaction, and IL17 signaling pathway (Figure 6G). These findings suggest that microglia may participate in ECM and synaptic remodeling in response to *RBFOX1* deficiency within the *APOE4* genetic background. To further investigate the role of microglia in synaptic remodeling, we performed co‐staining of neuronal marker TUJ1 and microglia marker IBA1, as well as CD68 and PSD95. The result revealed that microglia made contact with neurons, suggesting their correlation with synaptic remodeling (Figures 6H–I). These data demonstrate that *RBFOX1* deficiency remodels synaptic architecture via microglia‐ECM interplay in *APOE4* organoids.

### 3.7 Neuronal Network Dysfunction in *APOE4_RBFOX1*‐KO Organoids Reflects AD Pathophysiology

**FIGURE 7:**
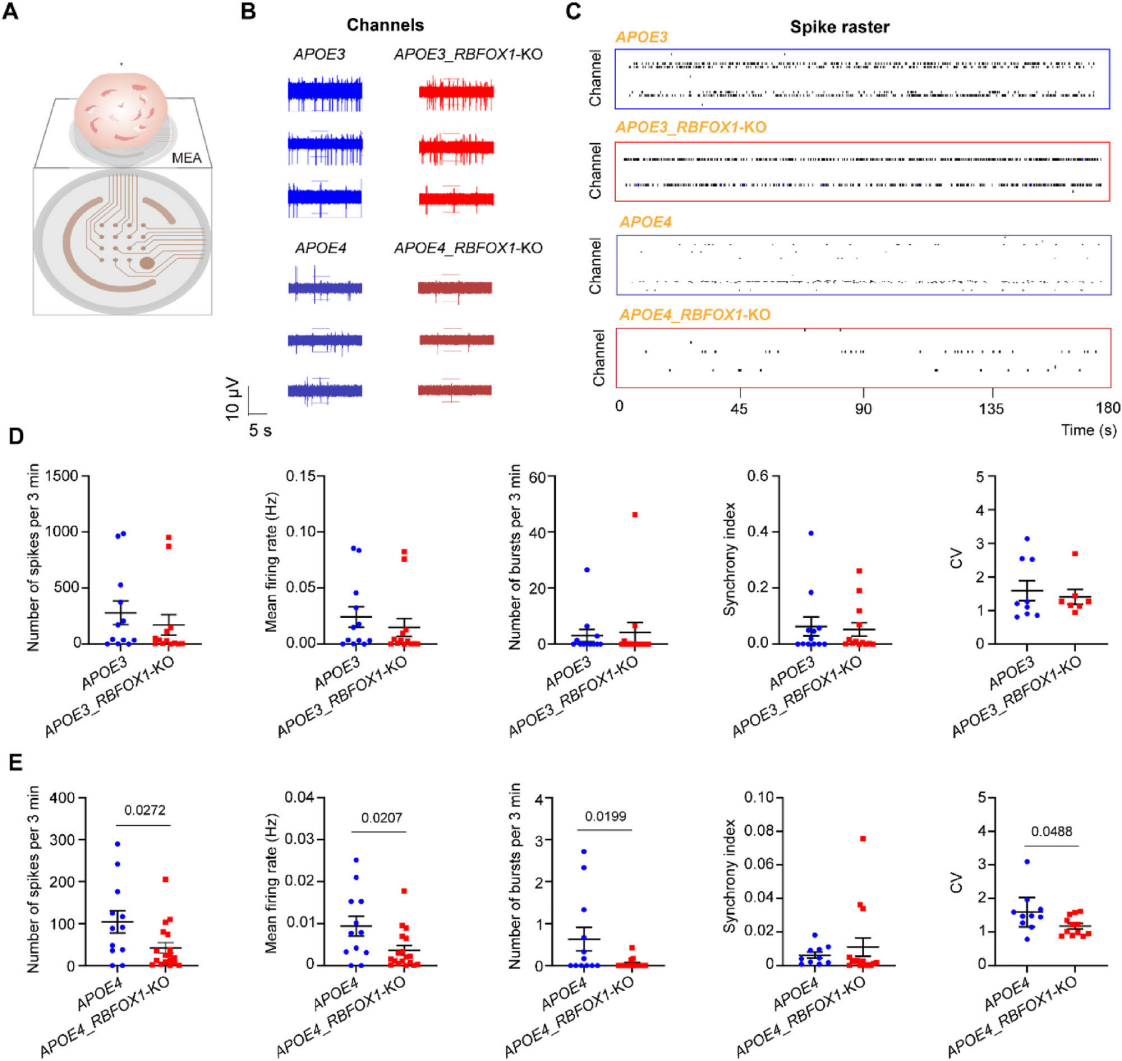
Neuronal network dysfunction in *APOE4_RBFOX1*‐KO organoids reflects AD pathophysiology. (A) Scheme of a cortical organoid plated on a MEA, (B) Representative electro‐traces recorded from individual organoids without (blue frame) and with (red frame) *RBFOX1*, (C) raster plots from selected regions. (D, E) MEA analyses for the number of spikes, mean firing rate, number of bursts, Synchrony index, and coefficient of variation (CV) among *RBFOX1*‐KO and control organoids at Day 60. Data represent the mean ± SEM, and were analyzed by a two‐tailed unpaired Student's *t*‐test (*n* ≥ 3 organoids; each data point represents one organoid; organoids were collected from three independent batches).

To assess functional consequences of synaptic remodeling, we recorded electrophysiological activity in organoids using a Maestro multi‐well microelectrode array (MEA, Axion Biosystems). Cerebral organoids were seeded on a 64‐electrode plate (Figure 7A), and key electrophysiological parameters were recorded noninvasively. The electrophysiological differences attributable to *RBFOX1* deficiency and *APOE* genotype were quantified. *APOE4* organoids lacking *RBFOX1* displayed reduced electrophysiological activity, including a decrease in spike and burst numbers, mean firing rate, and coefficient of variation (CV), while no significant differences were detected between *APOE3* and *APOE3*_*RBFOX1*‐KO organoids (Figures 7B–E).

This genotype‐dependent dysfunction is consistent with the observed synaptic protein dysregulation (Figures 6A–D) and microglia‐mediated neuroinflammation (Figure 2). The combined effects of tau hyperphosphorylation, lipid accumulation, and synaptic remodeling in *APOE4_RBFOX1*‐KO organoids collectively mirror the electrophysiological deficits observed in AD patients.

## 4 Discussion

*RBFOX1*, a known risk factor for neuropsychiatric disorders including epilepsy and autism [13, 14, 15], has recently emerged from GWAS analyses as a potential AD susceptibility gene [7, 8, 9]. Our study provides the first experimental evidence that *RBFOX1* deficiency exacerbates core AD pathologies: tauopathy, inflammatory responses, lipid accumulation, and impaired neuronal electrophysiological activity, specifically in *APOE4* cerebral organoids. These findings position *RBFOX1* as a critical genetic modifier that interacts with *APOE4* to exacerbate AD‐related pathogenic processes, offering mechanistic insights into the polygenic nature of sporadic AD.

The exacerbated tauopathy in *APOE4*_*RBFOX1*‐KO organoids may stem from dual mechanisms: (1) Increased total Tau protein levels (Figures 4E–F) and (2) the alternative splicing of *GSK3β* transcripts lacking exons 9/11 (Supplementary Figure S8), which encode isoforms reported to enhance Tau phosphorylation [93, 94]. Together, these observations suggest RBFOX1 regulates Tau metabolism via splicing‐dependent and splicing‐independent pathways; the precise molecular cascade requires further validation. Notably, the absence of Aβ pathology changes (Supplementary Figure S7) underscores RBFOX1's selective role in tauopathy, distinguishing it from APOE4's broader amyloidogenic effects.

It is noteworthy that we observed an apparent contradiction between the MEA results (Figure 7) and synaptic morphology analysis (Figures 6A–C) in *APOE4*_*RBFOX1*‐KO human brain organoids: MEA revealed reduced electrophysiological activity, whereas immunofluorescence staining demonstrated increased expression of presynaptic (Synapsin1) and postsynaptic (PSD95) proteins. While this may appear paradoxical, structural and functional synaptic alterations do not necessarily align. Indeed, elevated synaptic protein expression may reflect maladaptive or pathological processes rather than functional enhancement. In our *APOE4*_*RBFOX1*‐KO organoid model, Tau hyperphosphorylation is a key pathological feature. p‐Tau reduces the dynamics of PSD condensates [95] and impairs NMDA receptor mobility and clustering, thereby contributing to synaptic dysfunction despite increased synaptic marker abundance. Similarly, PSD95 phosphorylation promotes its accumulation at synapses [96], but excessive phosphorylation is linked to early synapse loss and cognitive deficits. Overexpression of PSD‐95 combined with NOS inhibition also disrupts axonal varicosity formation and multi‐synaptic connectivity [97], further illustrating that higher PSD95 levels do not invariably enhance network activity. Finally, human brain organoids are dynamic 3D systems in which synaptic protein expression and secretion fluctuate across developmental stages [98]. Such temporal dynamics may contribute to the observed dissociation between structural markers and functional outcomes. Taken together, these considerations suggest that synaptic molecular alterations and network activity in our model are related in a complex, non‐linear manner, highlighting an important direction for future investigation.

A pivotal finding is the exclusive spontaneous generation of microglia (about 5% of cells) in *APOE4*_*RBFOX1*‐KO organoids, recapitulating yolk sac‐derived microgliogenesis through conserved mesodermal priming (TGFβ/WNT activation) and EMP/MP differentiation (Figure 3). Unlike co‐culture models requiring exogenous microglial progenitors [30, 31, 32, 33], our system achieves autonomous microglial self‐organization, mirroring in vivo spatial distribution and DAM activation (Figures 1, 2). This advance addresses a major limitation of conventional organoids and provides a physiologically relevant platform to study neuron‐glia interactions. Unlike prior protocols that induced microglia through mesodermal retention via culture modifications (e.g., reduced heparin and delayed Matrigel embedment as shown by Ormel et al. [42]), our model demonstrates that the *APOE4* genotype combined with *RBFOX1* knockout is sufficient to induce microglia within a standardized cerebral organoid protocol. This suggests a distinct, genetically driven mechanism of microglial induction, potentially relevant to AD modeling. Meanwhile, our human brain organoid model with integrated microglia surpasses existing co‐culture‐derived systems by offering a platform to study mesodermal development [42], synaptic formation, and electrophysiological activity [33, 99]. It additionally models inflammatory response regulation [33] and AD pathology, establishing itself as a versatile tool for neuroscience and disease research.

We explored the mechanisms underlying spontaneous microglial generation. In organoids at Day 5, Day 7, and Day 10, we found that microglial production is likely associated with mesodermal development in early‐stage organoids. Knockout of *RBFOX1* prolonged the persistence of the mesodermal layer, especially TGFβ/WNT signaling, allowing EMPs to mature in parallel with organoid development (Figures 3B‐C). Specifically, we observed that rescue of *RBFOX1* expression in *APOE4_RBFOX1‐*KO organoids restored the expression of TGFβ genes to levels comparable to those in *APOE4* organoids (Supplementary Figure S5). This result demonstrates that RBFOX1 regulates the TGFβ pathway involved in microglial production. Additionally, we performed alternative splicing analysis on organoids at four time points (Day 5, Day 7, Day 10, and Day 60). In *APOE4_RBFOX1‐KO vs APOE4* organoids, we identified altered splicing of key TGFβ pathway molecules [100] (*LTBP4*, *SAMD4B*, and *AKT2*) at Day 7 (Supplementary Table S8), as well as splicing changes in *TGFBR2* and *SMAD1* at Day 60 (Supplementary Table S9). Literature reports [101] that Rbfox/LASR disruption alters splicing of TGFβ pathway‐related genes in mice (e.g., *Mapk2k4* [100] and *Fn1* [102]). Therefore, we hypothesize that RBFOX1‐containing complexes modulate TGFβ pathway genes, and their disruption in an APOE4 context may perturb TGFβ pathway homeostasis, thereby bias lineage specification toward microglial fate. This model highlights the critical interplay between RBFOX1‐mediated regulation, APOE4‐associated pathogenic traits, and TGFβ signaling in AD pathogenesis, underscoring the need for deeper mechanistic granularity to dissect the molecular basis of this tripartite crosstalk.

Despite APOE4 being the strongest genetic risk factor for AD, most carriers (>75%) escape clinical AD [103], emphasizing the necessity of co‐occurring risk factors. Our data reveal that *RBFOX1* deficiency acts synergistically with *APOE4*, amplifying p‐Tau levels (1‐5‐fold), neuroinflammation, and lipid accumulation—a pathological constellation that mirrors severe human AD. Crucially, this synergy enables microglial integration into the AD phenotype, addressing a longstanding gap in modeling glial contributions to neurodegeneration. While our findings primarily focus on AD, the observed interactions between *RBFOX1* deficiency and the *APOE4* allele in microglial activation suggest potential relevance to other neurodevelopmental disorders, where microglial involvement may also play a significant role in disease progression. Future studies exploring these connections could provide valuable insights into how these genetic factors influence both neurodegenerative and neurodevelopmental conditions.

In summary, by integrating *APOE4* genotype, *RBFOX1* deficiency, and endogenous microgliogenesis into a single model, we establish a human‐relevant system that recapitulates multifaceted aspects of AD pathophysiology. This platform helps bridge critical gaps in understanding genotype‐dependent glial‐neuronal interactions, paving the way for mechanistic discovery and therapeutic screening. However, several key limitations should be acknowledged, including: (1) Mechanistic granularity: The molecular basis of RBFOX1‐APOE4 crosstalk in mesodermal priming and DAM activation requires deeper interrogation. Furthermore, our current human brain organoids are derived from embryonic stem cell lines, which exhibit limited genetic diversity. Thus, utilizing patient‐specific iPSC libraries to generate *RBFOX1*‐knockout brain organoids with greater genetic heterogeneity would facilitate investigation into personalized AD pathological mechanisms. (2) In vivo validation and recapitulation of aging: Aging is a significant risk factor for AD pathogenesis [104]. Our current culture system produces organoids at a 2‐month developmental stage; despite reaching relative maturity, they inadequately simulate human brain aging, which underscores the need to optimize cultivation systems for longer‐term organoid development. Although some teams have cultivated human brain organoids for 170 days [105] or even 200 days [106],—a duration that allows observation of aging‐related protein aggregation,—these systems still cannot replicate the human brain's multi‐decadal aging process [106]. Future investigations using animal models may clarify how these mechanisms operate in the context of aging. Additionally, the proposed role of RBFOX1 in microglial lineage specification requires validation in animal models. Previous studies in mice have shown that *Rbfox1* deletion leads to neurodevelopmental and neurobehavioral abnormalities, including epilepsy [11] and autism‐like phenotypes [14, 107], highlighting its critical role in neuronal excitability and synaptic regulation. However, the specific role of RBFOX1 in cognitive function and memory within the context of AD has not been addressed in vivo. To bridge this gap, we are currently generating *Rbfox1* knockout mice on an AD‐prone background to test whether Rbfox1 loss exacerbates AD‐related pathology and cognitive decline. (3) Therapeutic relevance: As a newly identified risk factor for AD, RBFOX1 represents a potential therapeutic target. Its pathological association with APOE4 may enable more precise treatment strategies for AD patients with an *APOE4* genetic background. Furthermore, the enhanced microglia generation observed in RBFOX1‐knockout human brain organoids provides a model system to comprehensively investigate multiple AD‐related pathological factors, including microglial activation, tau phosphorylation, inflammatory responses, lipid accumulation, and aging processes, thereby facilitating more rational and effective decision‐making in AD drug development. However, the current model has several considerations that require attention: First, whether restoring RBFOX1 function can rescue APOE4‐associated pathologies remains untested. Second, the risk profile of RBFOX1 appears strongly dependent on APOE4 status, restricting its therapeutic applicability in non‐APOE4 carriers. Third, the absence of a vascular system in this model limits its utility for studying blood‐brain barrier involvement in AD pathogenesis. (4) AD is a multifactorial disorder involving complex interactions between genetic, epigenetic, and environmental factors. The KO model may overlook polygenic risk contributions identified in human GWAS, even after accounting for RBFOX1 knockout in the context of the APOE4 genotype, suggesting that there is an urgent need for more bold and innovative methodological approaches (e.g., polygenic risk score integration, combinatorial genetic editing, or multi‐omics modeling) to recapitulate the full complexity of AD pathogenesis and advance translational research.

## Author Contributions

R. Z. conceived the study. R. Z., Y. H., and B. Z. designed the experiments. B. Z., J. Z., Y. Q., H. Z. and H. G. performed the experiments. B. Z., J. Z., C. S. and R. Z. analyzed the data. B. Z., Y. H., and R. Z. wrote the manuscript. B. Z., Q. H., J. L., J. C., J. Y. C., J. X. and Y. H. edited the manuscript. R. Z., Y. H., B. Z., and T. K. participated in proposing modifications and contributed to the revision of the paper. All authors reviewed the manuscript.

## Conflicts of Interest

The authors declare no conflicts of interest.

## Supporting information




**Supporting File 1**: exp270160‐sup‐0001‐SuppMat.pdf.

**Supporting File 2**: exp270160‐sup‐0002‐TableS1.docx.

**Supporting File 3**: exp270160‐sup‐0003‐TableS2.docx.

**Supporting File 4**: exp270160‐sup‐0004‐TableS3.xlsx.

**Supporting File 5**: exp270160‐sup‐0005‐TableS4.xlsx.

**Supporting File 6**: exp270160‐sup‐0006‐TableS5.xlsx.

**Supporting File 7**: exp270160‐sup‐0007‐TableS6.xlsx.

**Supporting File 8**: exp270160‐sup‐0008‐TableS7.xlsx.

**Supporting File 9**: exp270160‐sup‐0009‐TableS8.xlsx.

**Supporting File 10**: exp270160‐sup‐0010‐TableS9.xlsx.

## Data Availability

Raw data for all sequencing experiments have been deposited at the Gene Expression Omnibus (GEO) under the accession number GSE292560. To review GEO accession GSE292560: Go to https://www.ncbi.nlm.nih.gov/geo/query/acc.cgi?acc=GSE292560, Enter token gzylskyirzwhjmf into the box.
