# Supplementary material for: RBFOX1 Dysfunction Unlocks APOE4‐Associated Microglial Genesis and Exacerbates Alzheimer's Pathology in Human Cerebral Organoids
Source: Exploration (Beijing). 2026 Apr 2;6(2):70160. doi: 10.1002/exp2.70160 (PMC13094536; doi:10.1002/exp2.70160)
Supplement: Supplementary file 1 — Supporting File 1: exp270160‐sup‐0001‐SuppMat.pdf. [file EXP2-6-70160-s006.pdf]

Figure S1

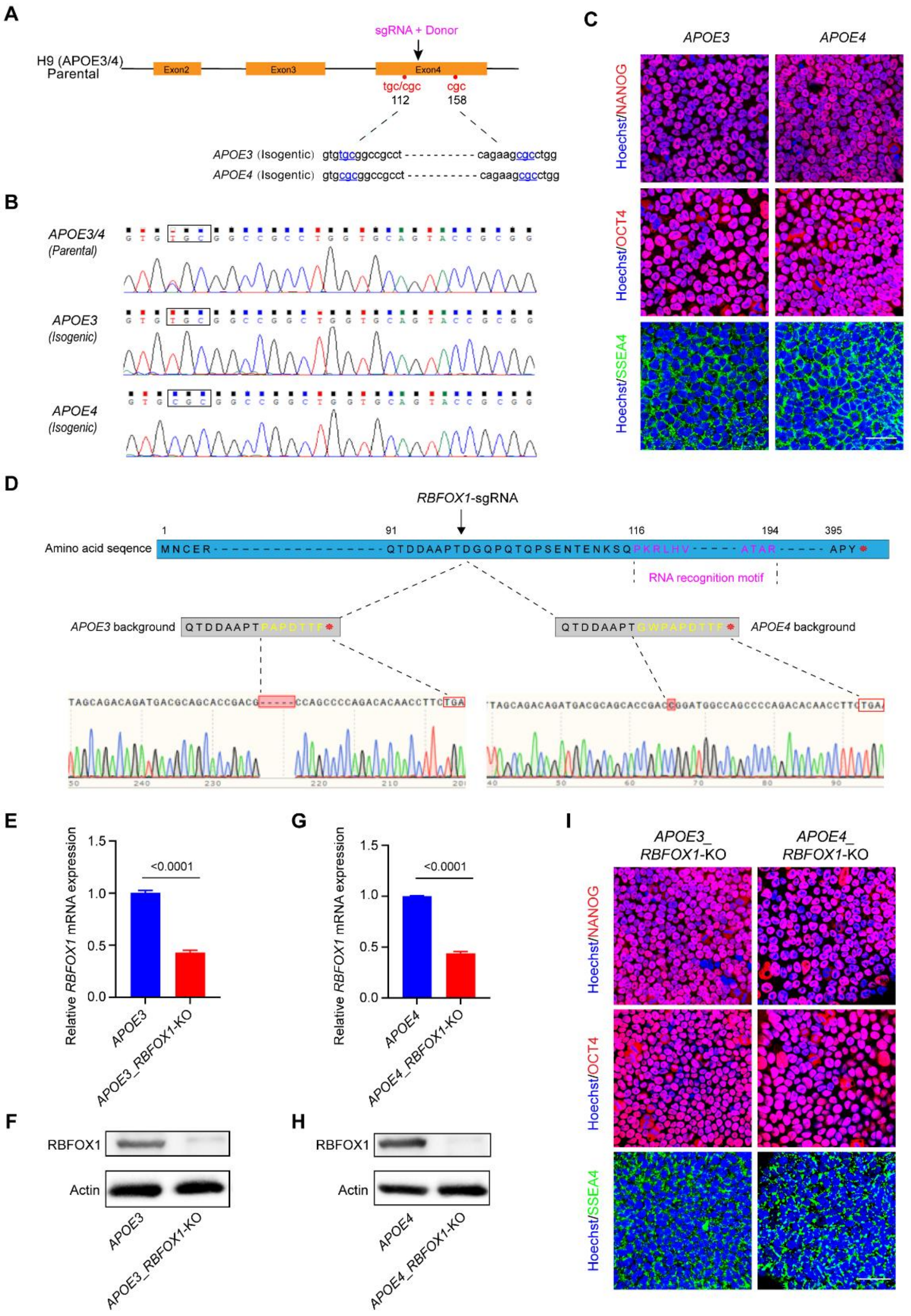

Figure S1. Characterization of isogenic *APOE3* or *APOE4* H9 cell lines with *RBFOX1* deletion. (A) Schematic of the strategy to generate hESC cell lines with homozygous *APOE3* or *APOE4* genotype from a APOE3/4 heterozygous H9 cell line by CRISPR/Cas9 technology. (B) Genotyping of single-cell clones homozygous for *APOE*. (C) Stemness identification in H9 cell clones homozygous for *APOE3* or *APOE4* with pluripotent markers

NANOG, OCT4 and SSEA4. (D) Generation of isogenic *APOE3* or *APOE4* H9 cell lines with *RBFOX1*-deletion via CRISPR/Cas9 technology and single-cell clone sequencing. (E-H) Verification of *RBFOX1* expression at the mRNA level by qPCR (E, G) (n = 3, data: mean ± SEM, and were analyzed by two-tailed unpaired Students *t*-test, and were collected from 3 independent experiments) and at the protein level by Western blot (F, H). (I) Identification of stemness in *APOE3_RBFOX1*-KO and *APOE4_RBFOX1*-KO H9 cell lines with pluripotency markers NANOG, OCT4 and SSEA4.

Figure S2

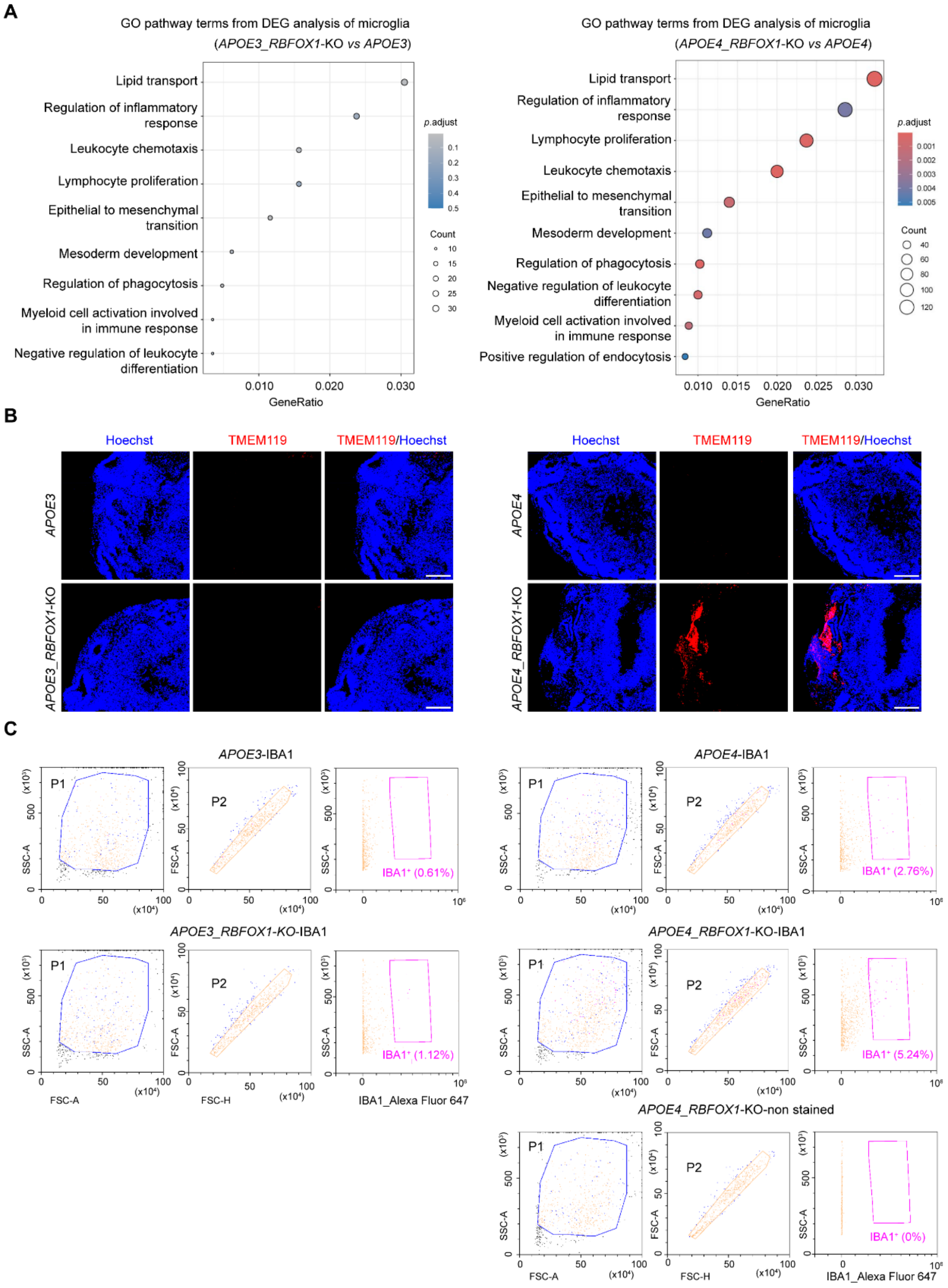

Figure S2. *RBFOX1* knockout induces the generation of microglia in human cerebral organoids carrying *APOE4* genotype at Day 60. (A) Gene Ontology (GO) analysis of differentially expressed genes (DEG) in microglia from *APOE3_RBFOX1-KO* vs *APOE3* and *APOE4_RBFOX1-KO* vs *APOE4* organoids at Day 60. (B) Low-magnification imaging showing the fluorescent signals of TMEM119 and their distribution within human brain

organoids at Day 60. Scale bars, 240 µm. (C) Flow cytometry analysis of IBA1 in Day 60 organoids.

Figure S3

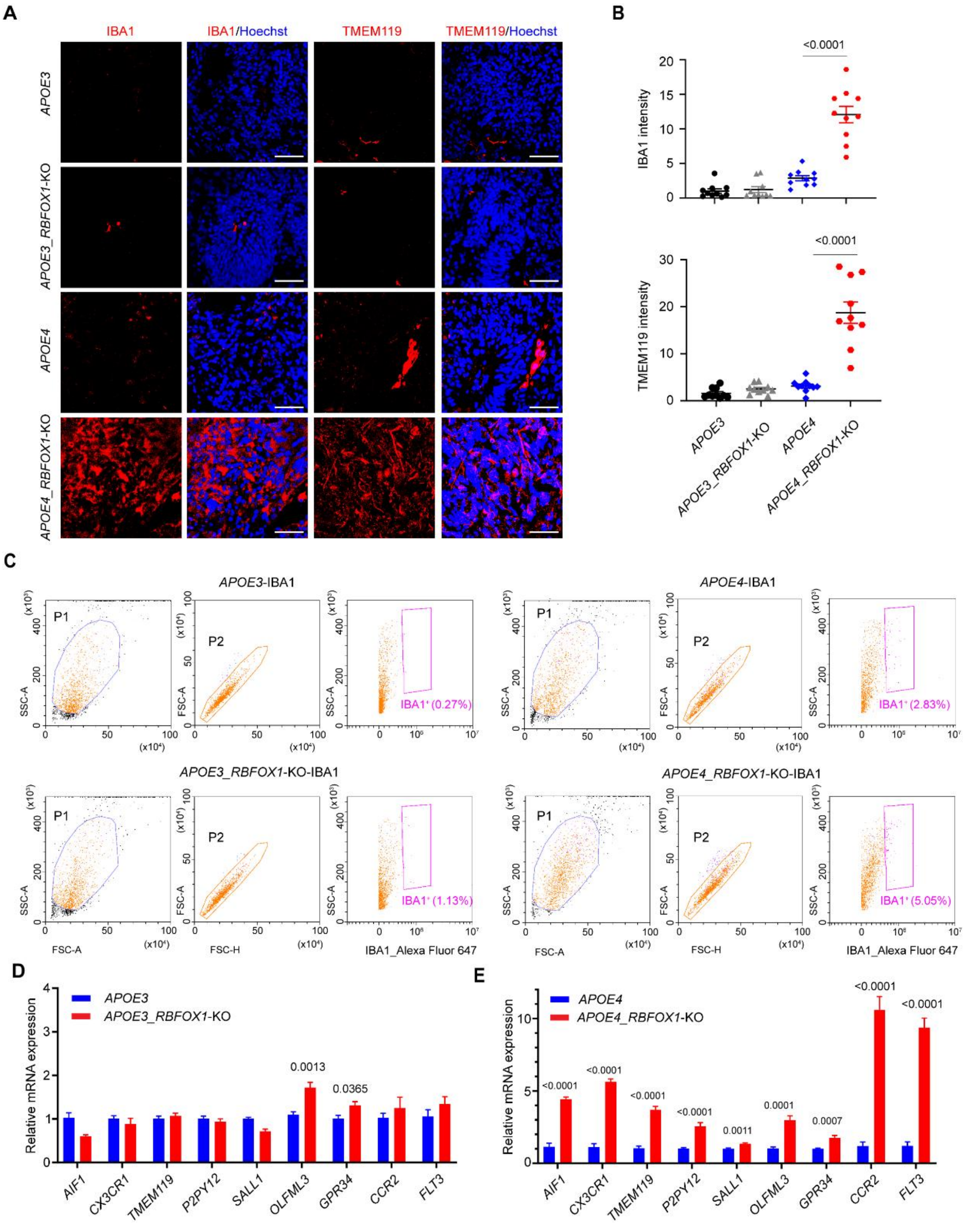

Figure S3. *RBFOX1* knockout induces the generation of microglia in human cerebral organoids carrying *APOE4* genotype at Day 44. (A) Representative immunofluorescent images showing microglial markers IBA1 and TMEM119 in organoids at Day 44, respectively. Scale bars, 50 μm. (B) Quantification of the IBA1 and TMEM119 average fluorescence intensity detected in (A). Data represent the mean ± SEM, and were analyzed by two-way ANOVA (n = 10; each data point represents one organoid; organoids were collected from three independent batches). (C) Flow cytometry analysis of IBA1-positive

cells confirms the presence of microglia in *APOE4*-organoids when *RBFOX1* was knocked out at Day 44. (D, E) qPCR analysis of relative mRNA expression of microglial-specific or microglia-enriched genes in organoids at Day 44. Genes in (D) were normalized to those in *APOE3*, and genes in (E) were normalized to *APOE4*. Data represent the mean ± SEM, and were analyzed by two-tailed unpaired Student's *t*-test (n = 3, data were collected from 3 independent experiments).

Figure S4

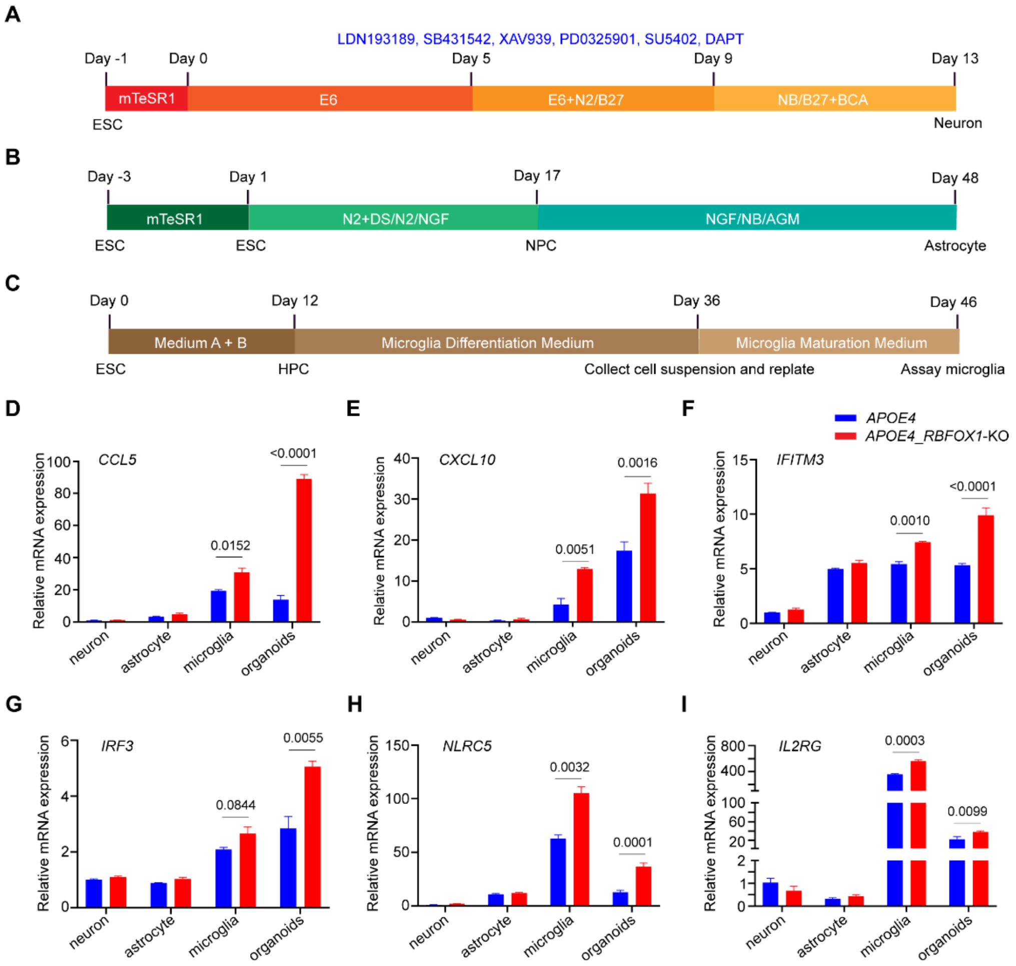

Figure S4. *RBFOX1* knockout has an effect on the development and function of multiple neuronal cells differentiated from the H9 cell line. (A-C) Schematic diagrams illustrating the differentiation of human embryonic stem cells (H9) into neurons, astrocytes, and microglia. (D-I) Differential expression of inflammatory factors *CCL5*, *CXCL10*, *IFITM3*, *IRF3*, *NLRC5*, and *IL2RG* in 2D culture of differentiated neurons, astrocytes, microglia, and organoids. Expression levels were normalized to those in *APOE4* neurons. Data: mean ± SEM, and were analyzed by two-tailed unpaired Student's *t*-test. (n = 3, data were collected from 3 independent experiments)

Figure S5

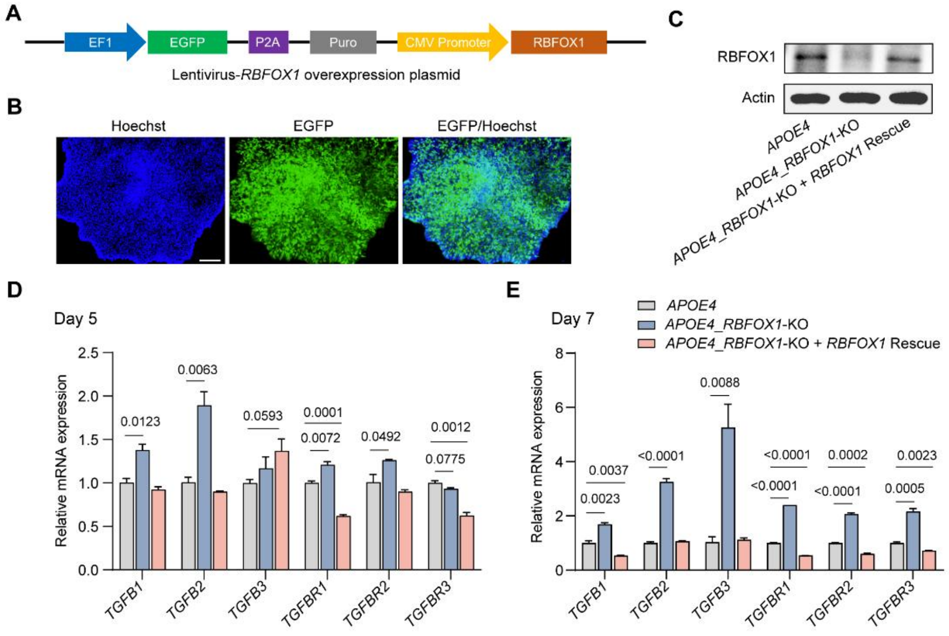

Figure S5. Rescue of RBFOX1 expression in *APOE4_RBFOX1-KO* organoids. (A) Schematic diagram of RBFOX1 overexpression plasmid. (B) Identification of RBFOX1 lentiviral plasmid infection by EGFP fluorescence signal. Scale Bar, 200 μm. (C) Detection of RBFOX1 overexpression in *APOE4_RBFOX1-KO* embryonic stem cell lines by western blot. (D, E) qPCR analyzing expression of key molecules in the TGFβ signaling pathway at early-stage organoid development (Day 5 and Day 7), data: mean ± SEM, and were analyzed by two-tailed unpaired Students *t*-test (The experiment collected 10 independent samples (biological replicates) at Day 5 and Day 7, which were pooled into a single sample after RNA extraction, with each sample subjected to three technical replicates (three qPCR wells) for qPCR analysis).

Figure S6

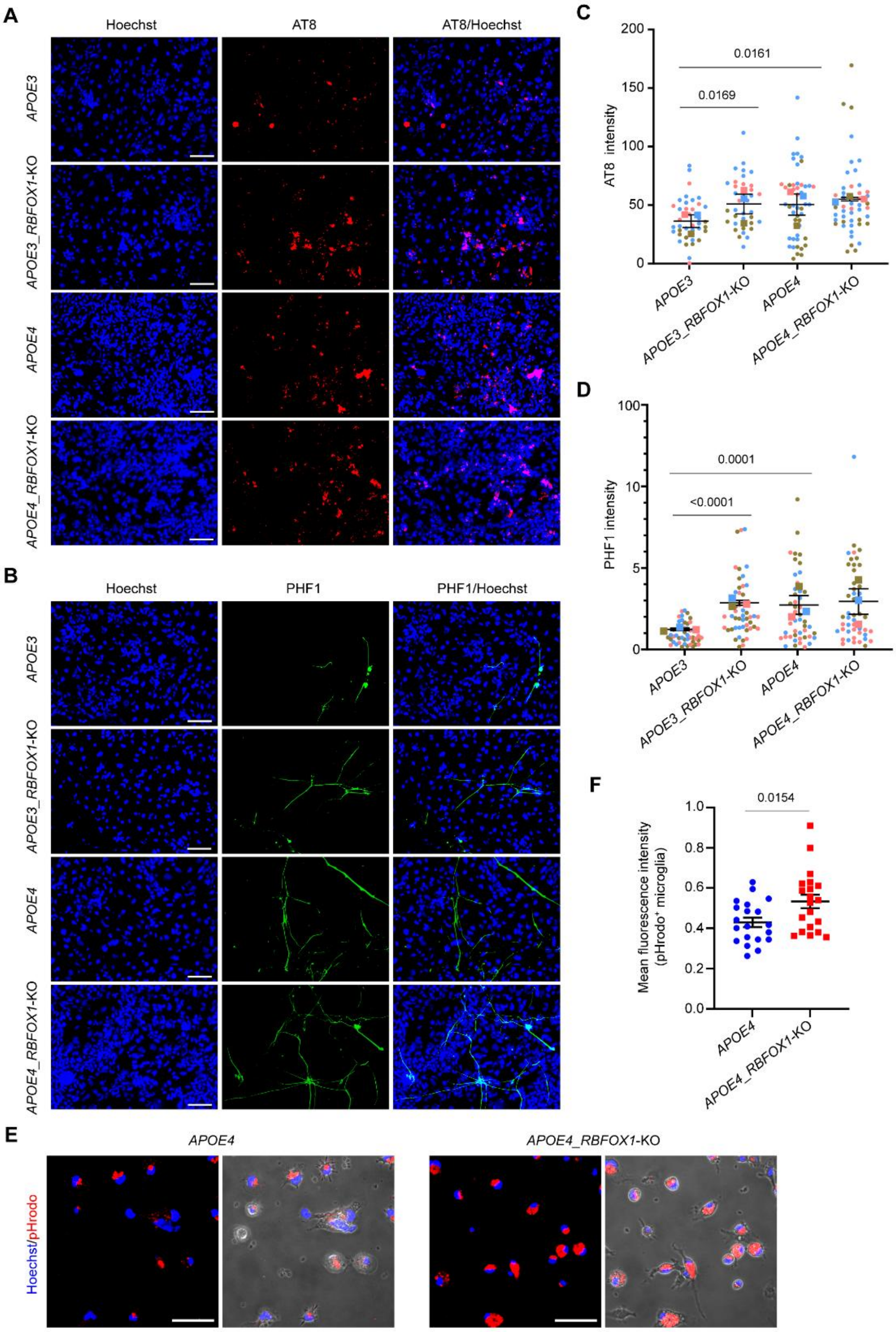

Figure S6. *RBFOX1* knockout exacerbates tau phosphorylation in H9-derived neurons. (A, B) Representative images showing p-tau staining by AT8 antibody and PHF1 antibody in neurons differentiated from H9 ESCs. Scale bars, 100 μm. (C, D) Quantification of the AT8 and PHF1 average fluorescence intensity shown in (A) and (B). Data represent mean ± SEM, and were analyzed by two-way ANOVA, (A solid square represents an independent experiment; a small dot denotes a microscopic field of view within each

experiment, with dots of the same color originating from the same independent experiment; n = 3, data were collected from 3 independent experiments). (E) Fluorescent images showing phagocytosis of pHrodo™-labeled BioParticles (red). (F) Quantification of fluorescence intensity from (E). Data represent the mean ± SEM, and were analyzed by two-tailed unpaired Student’s *t*-test. (n =20, each data point represents a random selected microscope field of view. Data were collected from 3 independent experiments)

Figure S7

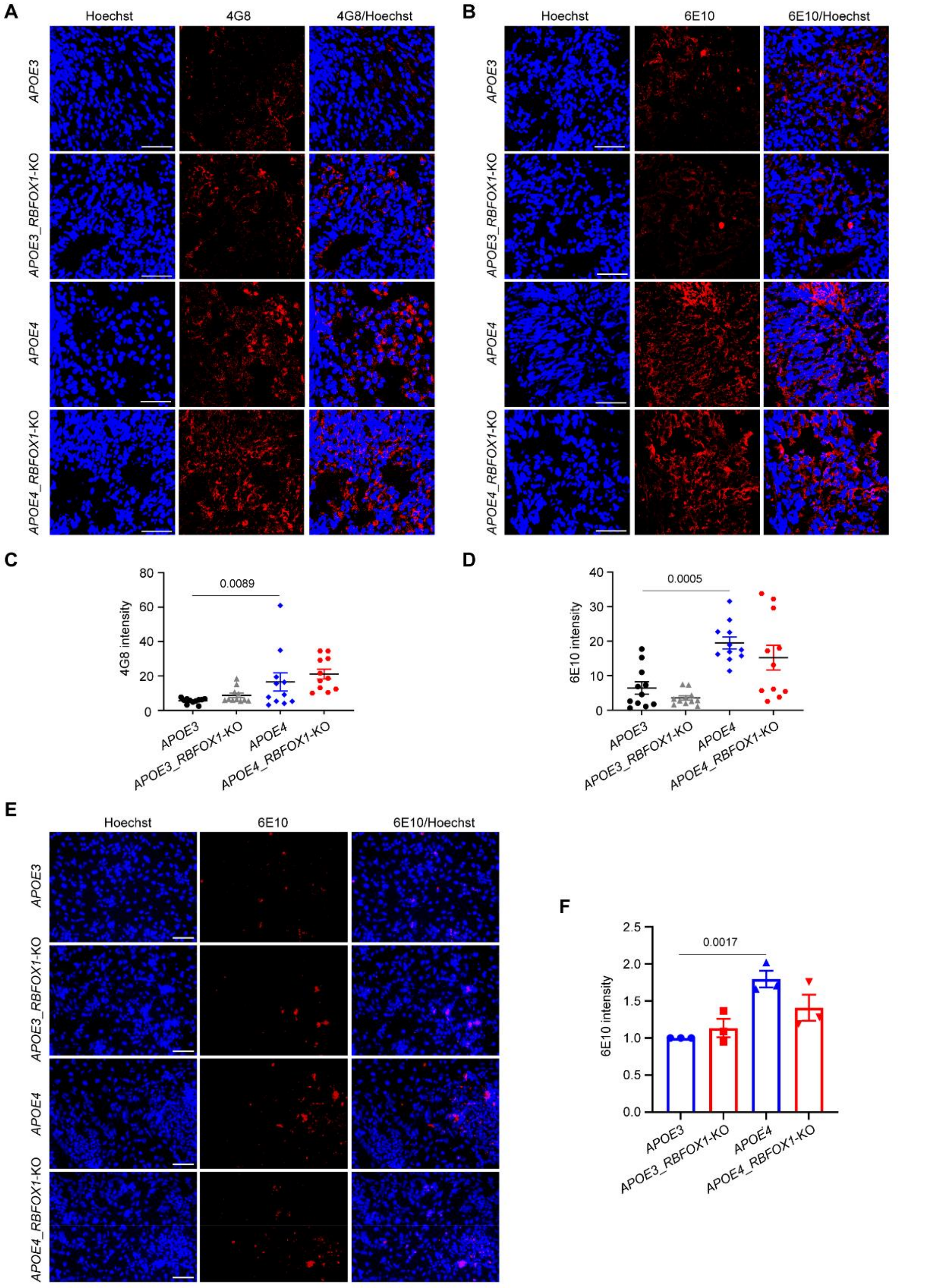

Figure S7. *RBFOX1* knockout does not affect Aβ deposition in cerebral organoids and neurons. (A, B) Representative images showing Aβ staining by 4G8 antibody and 6E10 antibody in organoids at Day 60. Scale bars, 100 μm. (C, D) Quantification of average fluorescence intensity of 4G8 and 6E10 in *RBFOX1*-KO and control organoids in (A) and (B). Data represent the mean ± SEM, and were analyzed by two-way ANOVA. (n = 11;

each data point represents one organoid; organoids were collected from three independent batches). (E) Representative images showing Aβ staining by 6E10 antibody in H9-derived neurons. Scale bars, 100 μm. (F) Quantification of 6E10 average fluorescence intensity in neurons shown in (E). Data: mean ± SEM, and were analyzed by two-way ANOVA. ($n = 3$; each dot represents one independent experiment).

Figure S8

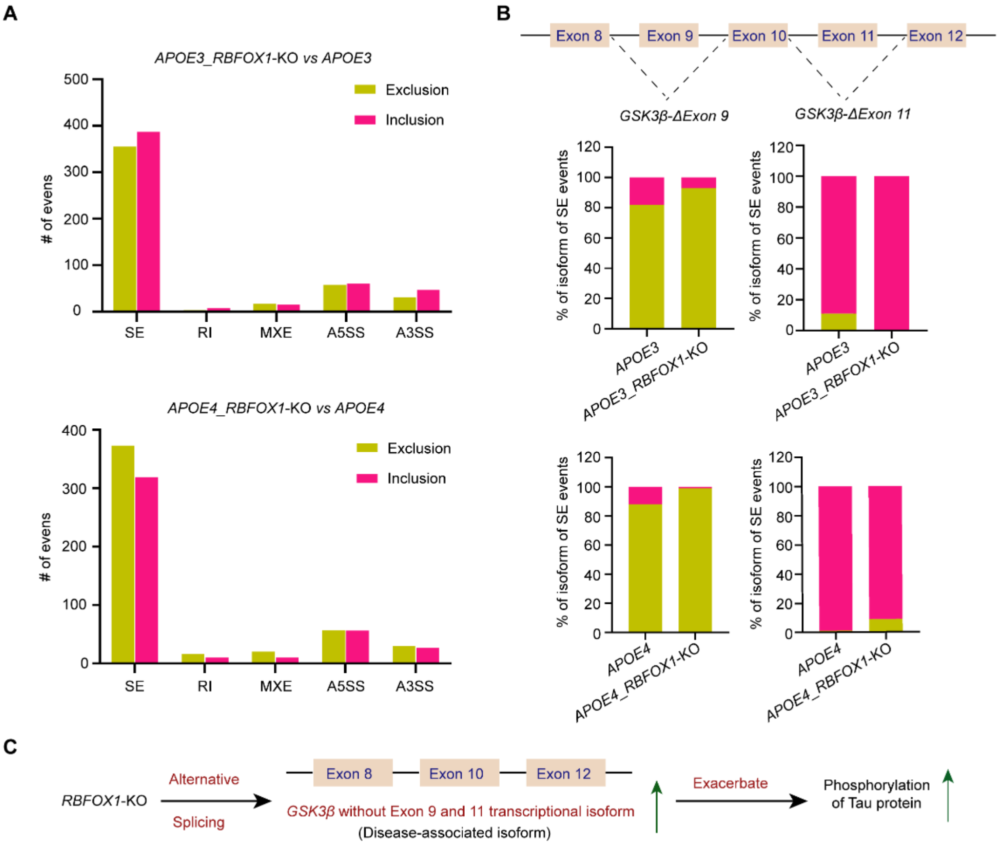

Figure S8. Analysis of disease-associated *GSK3β* transcripts, known for exacerbating tau phosphorylation. (A) Quantification of the different types of alternative splicing events regulated by *RBFOX1* knockout in *APOE3* or *APOE4* organoids at Day 60. SE: Skipped exon, RI: Retained intron, MXE: Mutually exclusive exon, A5SS: Alternative 5' splice site, A3SS: Alternative 3' splice site. (B) The altered ratios of disease-associated *GSK3β* transcripts, left panel, alternative splicing of exon 9 in *GSK3β* transcripts; right panel, alternative splicing of exon 11 in *GSK3β* transcripts. (C) Schematic proposal illustrating that with *RBFOX1* knockout promotes the expression of *GSK3β* transcripts lacking exon 9 and exon 11, which is associated with increased tau phosphorylation.
