# Supplementary material for: RBFOX1 Dysfunction Unlocks APOE4‐Associated Microglial Genesis and Exacerbates Alzheimer's Pathology in Human Cerebral Organoids
Source: Exploration (Beijing). 2026 Apr 2;6(2):70160. doi: 10.1002/exp2.70160 (PMC13094536; doi:10.1002/exp2.70160)
Supplement: Supplementary file 2 — Supporting File 2: exp270160‐sup‐0002‐TableS1.docx. [file EXP2-6-70160-s002.docx]

| sgRNA for APOE | Sequence (5' to 3') |
| --- | --- |
| APOE-gRNA-F | CACCGCCTCGCCGCGGTACTGCACC |
| APOE-gRNA-R | AAACGGTGCAGTACCGCGGCGAGGC |

Supplementary Table S1 CRISPR/Cas9 sequences for generation of isogenic H9 lines

| Donor for APOE genotype | Sequence (5' to 3') |
| --- | --- |
| APOE3-Donor | CTGTCCAAGGAGCTGCAGGCGGCGCAGGCCCGGCTGGGCGCGGACATGGAGGACGTGTGCGGCCGGCTGGTGCAGTACCGCGGCGAGGTGCAGGCCATGCTCGGCCAGAGCACCGAGGA |
| APOE4-Donor | CTGTCCAAGGAGCTGCAGGCGGCGCAGGCCCGGCTGGGCGCGGACATGGAGGACGTGCGCGGACGCCTGGTGCAGTACCGCGGCGAGGTGCAGGCCATGCTCGGCCAGAGCACCGAGGA |

| sgRNA for RBFOX1 | Sequence (5' to 3') |
| --- | --- |
| RBFOX1-gRNA-F | CACCGATGACGCAGCACCGACGGA |
| RBFOX1-gRNA-R | AAACTCCGTCGGTGCTGCGTCATC |
