# Supplementary material for: RBFOX1 Dysfunction Unlocks APOE4‐Associated Microglial Genesis and Exacerbates Alzheimer's Pathology in Human Cerebral Organoids
Source: Exploration (Beijing). 2026 Apr 2;6(2):70160. doi: 10.1002/exp2.70160 (PMC13094536; doi:10.1002/exp2.70160)
Supplement: Supplementary file 3 — Supporting File 3: exp270160‐sup‐0003‐TableS2.docx. [file EXP2-6-70160-s001.docx]

Supplementary Table S2 qPCR primers sequences

qPCR primers for microglia marker primers

| AIF1-F | AGACGTTCAGCTACCCTGACTT |
| --- | --- |
| AIF1-R | GGCCTGTTGGCTTTTCCTTTTCTC |
| P2RY12-F | TTTGTGTGTCAAGTTACCTCCG |
| P2RY12-R | CTGGTGGTCTTCTGGTAGCG |
| TREM2-F | CTGCTCATCTTACTCTTTGTCAC |
| TREM2-R | CAGTGCTTCATGGAGTCATAGG |
| GPR34-F | CCTGATGTCCAGTAACATTCGC |
| GPR34-R | CATGCAGGGAGTATCCTGGT |
| CABLES1-F | AACGAGAGATGCGGAAGC |
| CABLES1-R | CGGTTCTGTTTGTTGAGTTTCC |
| BHLHE41-F | CTGCTTCCTCTCGCCTTC |
| BHLHE41-R | CGGGTACAGATACTTCTCCAG |
| OLFML3-F | CTGGTGGAGGTGGTGAGA |
| OLFML3-R | GATGTAGGTGTCTGCTGTCAAG |
| PROS1-F | TTGCACTTGTAAACCAGGTTGG |
| PROS1-R | CAGGAACAGTGGTAACTTCCAG |
| TMEM119-F | CTTCCTGGATGGGATAGTGGAC |
| TMEM119-R | GCACAGACGATGAACATCAGC |
| SALL1-F | CATATTGGCAGCGGCAGTTAC |
| SALL1-R | CTGGTGAGGACGATGATGAGAC |
| CX3CR1-F | CTTACGATGGCACCCAGTGA |
| CX3CR1-R | CAAGGCAGTCCAGGAGAGTT |
| CLEC12A-F | CAGGAACCTCTCCACCACACT |
| CLEC12A -R | CTGGCATTCTGAGCAGCACAG |
| MS4A7-F | GACCTGAGCAGCTTGACCT |
| MS4A7-R | CCACAATGTTGAGAGGCAGTC |
| CCR2-F | CCTGCCGCTGCTCATCAT |
| CCR2-R | TCACTGCCCTATGCCTCTTCT |
| MS4A3-F | GCATGGTGTCTCTACTGCTGATT |
| MS4A3-R | GCATTGCACCACATGGCTATG |
| FLT3-F | CCCTTCCCTTTCATCCAAGACAAC |
| FLT3-R | CGGTCACCTGTACCATCTGTAGC |
| hActin-F | GCTCCTCCTGAGCGCAAG |
| hActin-R | CATCTGCTGGAAGGTGGACA |

qPCR primers for mesoderm, EMP, MP, TGFB pathway and Wnt pathway

| MESP1-F | GAAGTGGTTCCTTGGCAGAC |
| --- | --- |
| MESP1-R | TCCTGCTTGCCTCAAAGTGT |
| MSGN1-F | TGCCCTGCACACCCTCCGGAATTA |
| MSGN1-R | TGCCGCGGTTAAGGAGGTCTGTGA |
| SNAI1-F | CTCGACCACTATGCCGCGCTCTTT |
| SNAI1-R | TCCCAGATGAGCATTGGCAGCGAG |
| HAND1-F | GTGAGAGCAAGCGGAAAAG |
| HAND1-R | GTGCGTCCTTTAATCCTCTTC |
| GATA6-F | CAGCAAGATGAACGGCCTCA |
| GATA6-R | TGACAGTTGGCACAGGACAA |
| BMP4-F1 | TGAAGCCCCCAGCAGAAGTGGT |
| BMP4-R1 | AGGACCGCAGGGCTCACATCAA |
| CHRD-F | TGCCCCCTTTTGGAGAGATG |
| CHRD-R | CAGTGAACAGTCATCCCGCT |
| NOTO-F | CAGTTACATCTGCCGAGGCT |
| NOTO-R | TCCTGACTCGGCATCATCAC |
| TBX6-F | AAGTACCAACCCCGCATACA |
| TBX6-R | TAGGCTGTCACGGAGATGAA |
| SFRP2-F | GTGTCCGAAAGGGACCTGAA |
| SFRP2-R | CAGATAGGGCGCGTTGATGT |
| LEFTY2-F | CGTGAGGGCCCAGTATGTAG |
| LEFTY2-R | AGCTCTGGCTGAACCTCTTTC |
| OTX2-F | CTGTTTGCCAAGACCCGGTA |
| OTX2-R | AAACCATACCTGCACCCTCG |
| LHX1-F | CCCCAGTTTGTCTCCGGATT |
| LHX1-R | ATTCTCGTTGCTACCCGCTT |
| TFAP2A-F | AGCTGCCAACGTTACCCTGCTCAC |
| TFAP2A-R | GGCAGGAAATTCGGTTTCGCACACG |
| DLX5-F | AGCGCCACCAACCAGCCAGAGAAA |
| DLX5-R | GTTTGTGTCAATCCCAGCGAGGCGG |
| GATA3-F | TCCTCCTCCTCTCTGCTCTTCGCT |
| GATA3-R | AGGGGCGACGACTCTGCAATTCT |
| CDH1-F | GCTGGAGATTA TCCGGACA |
| CDH1-R | ACCTGAGGCTTTGGATTCCT |
| TBXT-F | ATAGTGAGAGTTGGGGGTCCA |
| TBXT-R | TCACCGCTATGAACTGGGTC |
| PDGFRA-F | CTCCTACGACAGCAGACAGG |
| PDGFRA-R | CTTTCCTTTGACGGTGGCCT |
| CD44-F | ATGGCCCAGATGGAGAAAGC |
| CD44-R | GGGAGGTGTTGGATGTGAGG |
| KIT-F | aggttgttgaggcaactgct |
| KIT-R | ccgttctgtcaaatgggcac |
| MYB-F | GTCAATGTCCCTCAGCCAGC |
| MYB-R | TCGCTTTTCCTTCTCAGGGTC |
| CD34-F | CAACGGTACTGCTACCCCAG |
| CD34-R | CAGGCTGGTACTTCCAAGGG |
| CD32-F | ctggaaggacaagcctctgg |
| CD32-R | actgtggtttgcttgtggga |
| CD36-F | TGCAAGTCCTGATGTTTCAGA |
| CD36-R | AATAGGTTGACCTGCAGCCG |
| ADGRE1-F | gcaggtgtcatggcttacct |
| ADGRE1-R | ccgttgagcagacagtggat |
| CSF1-F | GCAGGAGTATCACCGAGGAG |
| CSF1-R | CACGAGGTCTCCATCTGACTG |
| IRF4-F | CTCTTCAAGGCTTGGGCACT |
| IRF4-R | CTCTTGTTCAAAGCGCACCG |
| TGFB1-F | CCGTGGAGGGGAAATTGAGG |
| TGFB1-R | TTGCAGTGTGTTATCCCTGCT |
| TGFB2-F | CAGTGGGAAGACCCCACATC |
| TGFB2-R | CAATAGGCCGCATCCAAAGC |
| TGFB3-F | AGCAGAATTCCGGGTCTTGC |
| TGFB3-R | CATCTGGCCGAAGGATCTGG |
| TGFBR1-F | GGCCCCTGAAGTTCTCGATG |
| TGFBR1-R | GCCCATTGCATAGATGTCAGC |
| TGFBR2-F | GTATCGCCAGCACGATCCCA |
| TGFBR2-R | GAAACTTGACTGCACCGTTGTT |
| TGFBR3-F | ATACAGGTTCCAGCCCTTGG |
| TGFBR3-R | ACACCACGATTTCAGGTCGG |
| WNT3-F1 | tgcaccaccatagatgacagc |
| WNT3-R1 | agtcacagccgcaaatggtg |
| DKK1-F | gcaccttggatgggtattcca |
| DKK1-R | gcacaacacaatcctgaggc |
| RSPO3-F | GTCCATGCACGAAGAAGGGA |
| RSPO3-R | TGTTGGGGGACACAGGTTAC |
| APELA-F | TCAGCGGACAGAGACCAGTT |
| APELA-R | GGTACTCGTGAATGGAGAGGC |
| CER1-F | TTTGGGAAATGCGGGTCTGT |
| CER1-R | GGTGAACTTGGCAGGCAAAC |
| MEF2C-F | GTCGGCTCAGTCATTGGCTA |
| MEF2C-R | CCTCCCATTCCTTGTCCTGG |
| SNAI2-F | CTGCGGCAAGGCGTTTTCCAGA |
| SNAI2-R | TGCAAATGCTCTGTTGCAGTGAGGG |
| CDH2-F | tcagtggcggagatcctact |
| CDH2-R | gtgactaacccgtcgttgct |

qPCR primers for inflammation factors

| CCL5-F | tcattgctactgccctctgc |
| --- | --- |
| CCL5-R | tactccttgatgtgggcacg |
| CX3CL1-F | gtggccatgttcacctacca |
| CX3CL1-R | cgggcaccaggacatatgaa |
| CXCL10-F | ctgtacgctgtacctgcatc |
| CXCL10-R | gcaatgatctcaacacgtggac |
| DHX58-F | acctgaggcgctacaatgac |
| DHX58-R | gtgacgtgctccctgtgata |
| EGR3-F | ccaggattaccaatcggcca |
| EGR3-R | gtaatagggggcgggttgac |
| FOSL2-F | gctcaggcagtgcattcatc |
| FOSL2-R | atgggttggacatggaggtg |
| HPRT-F | aggccatcacattgtagccc |
| HPRT-R | tgtaatccagcaggtcagcaa |
| IFIT3-F | ggctctggaagaaaagccca |
| IFIT3-R | agctcaatggcctgcttcaa |
| IFITM3-F | tgtccaaaccttcttctctcctg |
| IFITM3-R | ggatgtggatcacggtggac |
| IRF3-F | tatgccctctggttctgtgtg |
| IRF3-R | cccgggccatttctaccaag |
| IRF7-F | ctcggaactgtgacacccc |
| IRF7-R | gcccaggtagatggtatagcg |
| ISG15-F | ggtggacaaatgcgacgaac |
| ISG15-R | tcgaaggtcagccagaacag |
| NLRC5-F | gtggaggttctccctcacct |
| NLRC5-R | cccttgccaagcagagtagg |
| NLRP3-F | ttacctgcgaggcaacactc |
| NLRP3-R | tgacgtgaggttgcagttgt |
| XAF1-F | acttccacgatggagaaagatgt |
| XAF1-R | ggcttgggctctgacatcaa |
| IL1B-F | ctgtacctgtcctgcgtgtt |
| IL1B-R | gggaactgggcagactcaaa |
| IL2RG-F | ggatgggcagaaacgctaca |
| IL2RG-R | ttgggtggctccattcactc |
| IL6-F | cactggcagaaaacaacctga |
| IL6-R | attttcaccaggcaagtctcct |
| IL11-F | gcaggtggctcttccctgaa |
| IL11-R | gcagccggtccagtcg |
| IL34-F | aggtggaatccgtgttgtcc |
| IL34-R | gccagtttaggacggagctt |
